# Pulmonary fibroblast-derived stem cell factor promotes neutrophilic asthma by augmenting IL-17A production from ILC3s

**DOI:** 10.1172/JCI187372

**Published:** 2025-07-17

**Authors:** Jheng-Syuan Shao, Alan Chuan-Ying Lai, Wei-Chang Huang, Ko-Chien Wu, Po-Yu Chi, Yao-Ming Chang, Ya-Jen Chang

**Affiliations:** 1 Taiwan International Graduate Program in Molecular Medicine, National Yang Ming Chiao Tung University and Academia Sinica, Taipei, Taiwan.; 2 Institute of Biomedical Sciences, Academia Sinica, Taipei, Taiwan.; 3 Division of Chest Medicine, Department of Internal Medicine, Taichung Veterans General Hospital, Taichung, Taiwan.; 4 Department of Post-Baccalaureate Medicine, College of Medicine, National Chung Hsing University, Taichung, Taiwan.; 5 School of Medicine, Chung Shan Medical University, Taichung, Taiwan.; 6 Graduate Institute of Medicine, College of Medicine, Kaohsiung Medical University, Kaohsiung, Taiwan.; 7 Institute of Translational Medicine and New Drug Development, China Medical University, Taichung, Taiwan.; 8 Department and Graduate Institute of Microbiology and Immunology, National Defense Medical Center, Taipei, Taiwan.

**Keywords:** Immunology, Inflammation, Asthma, Innate immunity

## Abstract

Group 3 innate lymphoid cells (ILC3s) have emerged as an important player in the pathogenesis of neutrophilic asthma. However, the regulatory mechanism supporting ILC3 responses in the lung remains largely unclear. Here, we demonstrated that stem cell factor (SCF) expression is significantly increased and positively correlated with IL-17A and MPO expression in asthmatic patients. Notably, we identified ILC3 as a major IL-17A–producing responder to SCF in the lung. In mice, SCF synergized with IL-1β/IL-23 to enhance pulmonary ILC3 activation and neutrophilic inflammation. Mechanistically, SCF promoted ILC3 proliferation and cytokine production. Transcriptomic analysis revealed that SCF treatment upregulated the genes related to proliferation and Th17 differentiation, associated with increased AKT and STAT3 signaling. In contrast, deficiency of SCF receptor c-Kit reduced ILC3 proliferation and IL-17A production, resulting in the amelioration of airway hyperreactivity (AHR) and neutrophilic inflammation in mouse neutrophilic asthma model. Furthermore, genetic deletion of SCF in fibroblasts revealed fibroblasts as the primary source of SCF for ILC3 activation in the lung. Moreover, administration of imatinib, a c-Kit inhibitor, alleviated LPS, air pollution or ovalbumin/LPS-induced AHR and neutrophilic inflammation. Our findings elucidated a positive modulatory role of SCF/c-Kit signaling in ILC3 responses during neutrophilic inflammation, offering a potential therapeutic target for neutrophilic asthma.

## Abstract 2

SCF is a positive regulator of ILC3 and neutrophilic inflammation, suggesting a potential target for alleviating neutrophilic asthma

## Graphical abstract

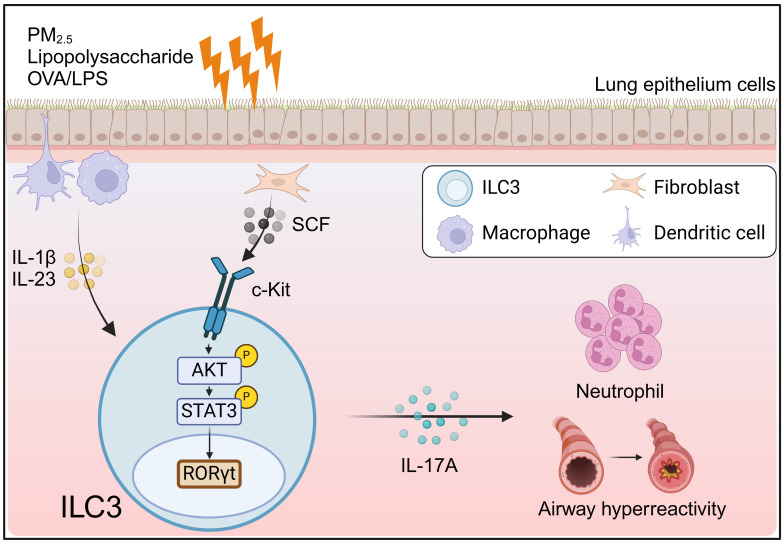

## Introduction

Asthma is a heterogeneous airway disease associated with airway inflammation, airway hyperreactivity (AHR) and respiratory symptoms, such as shortness of breath, coughing and wheezing (1). It is estimated to afflict about 339 million people worldwide, leading to huge healthcare and economic burdens (2). Among the total asthmatic cases, approximately 30% were neutrophilic asthma (3–5). Several factors have been identified as potential triggers for neutrophilic asthma, including bacterial infections, air pollution, and smoking (6–8). Notably, neutrophilic asthma is associated with more severe asthma, longer duration of asthma, worse lung function impairment, and has a greater incidence of hospitalization (7, 9–14). However, current research and biologics development for asthma mainly focused on eosinophilic asthma, leaving neutrophilic asthma with a lack of effective treatment options (8, 15–19). Therefore, there is an urgent need to discover novel therapeutic strategies for neutrophilic asthma.

Group 3 innate lymphoid cells (ILC3s), a group of lymphocytes that lack antigen receptor, have emerged as a key player to fine tune host defense against pathogens and maintain immune homeostasis in the mucosal immunity. ILC3s express RAR-related orphan receptor γ t (ROR-γt) and secrete IL-17 and IL-22 in response to IL-1β and IL-23 (20, 21). Functionally, ILC3-derived IL-17 and IL-22 could induce neutrophil recruitment and maintain epithelial barrier, respectively (22). IL-17A has also been shown to contribute to mucus production and AHR (23, 24), and is associated with neutrophilic inflammation and severe asthma (9, 25–28). In humans, ILC3s are numerically abundant ILC subsets in lung (29, 30). Recently, ILC3s were implicated in the pathophysiology of neutrophilic asthma. In particular, Kim et al. has shown that ILC3s play important roles in the induction of AHR in an obese neutrophilic asthma mouse model, and IL-17A is required for the development of AHR (31). Moreover, ILC3s were found to be increased in the BALF of patients with severe asthma (31). Studies also demonstrated that cigarette smoking asthma patients had increased blood ILC3s, which positively correlate with circulating neutrophils and asthma severity (32). In addition, noneosinophilic asthma patients were reported to have higher frequency and numbers of blood ILC3s, which may contribute to dexamethasone insensitivity through the production of neutrophil chemoattractants (33). Moreover, ILC3s were found to be increased and play a crucial role in LPS- (34) and ovalbumin (OVA)/LPS-induced neutrophilic asthma mouse model (35). However, the regulatory mechanism supporting lung ILC3 function during neutrophilic inflammation remains largely unclear.

Stem cell factor (SCF) is well known for its role in the survival, proliferation, and migration of haematopoietic stem cells, mast cells and melanocytes (36, 37). SCF binds to its receptor, c-Kit, and activates downstream signaling pathways, including Jak/Stat, Ras/Erk, and PI3k/Akt (36, 38). Interestingly, asthmatic patients are reported to have higher SCF expression in airway, serum, and blood (39–41). And, the concentrations of SCF is positively correlated with the severity of asthma (40). In a mouse model, SCF was shown to induce asthma in WT mice, which was dependent on mast cell activation (42, 43). And, the inhibition of SCF could reduce lung inflammation in allergic mice (44). Recently, the therapeutic potential of c-Kit inhibition in severe asthma patients has gained significant attention. Cahill et al. has conducted a Phase 2 trial to evaluate the effects of c-Kit inhibition with imatinib in severe asthma patients (45), and The Precision Interventions for Severe and/or Exacerbation-Prone Asthma Network (PrecISE) also launched a Phase 2 trial, which includes imatinib for treating severe asthma (46). Nevertheless, these studies primarily focused on the impact of c-Kit inhibition on mast cells, leaving the effects of c-Kit inhibition on other immune cells largely unclear. Since both ILC2s and ILC3s express c-Kit on their surface (20, 47), this suggests a potential role of SCF/c-Kit signaling in ILC2- and ILC3-mediated asthma. Indeed, a study showed that in ILC2-mediated mouse asthma model, inhibition of SCF/c-Kit signaling reduced the number and cytokine production of ILC2s and mitigated allergic airway inflammation (48). However, the precise role of SCF/c-Kit signaling in ILC3 responses and its impact on neutrophilic inflammation remain unexplored.

Given the increase of SCF in asthmatics and the expression of c-Kit on ILC3s, we hypothesized that SCF/c-Kit signaling may play important roles in regulating ILC3 function and neutrophilic inflammation.

## Results

### SCF expression is increased in asthmatic patients.

**Figure 1:**
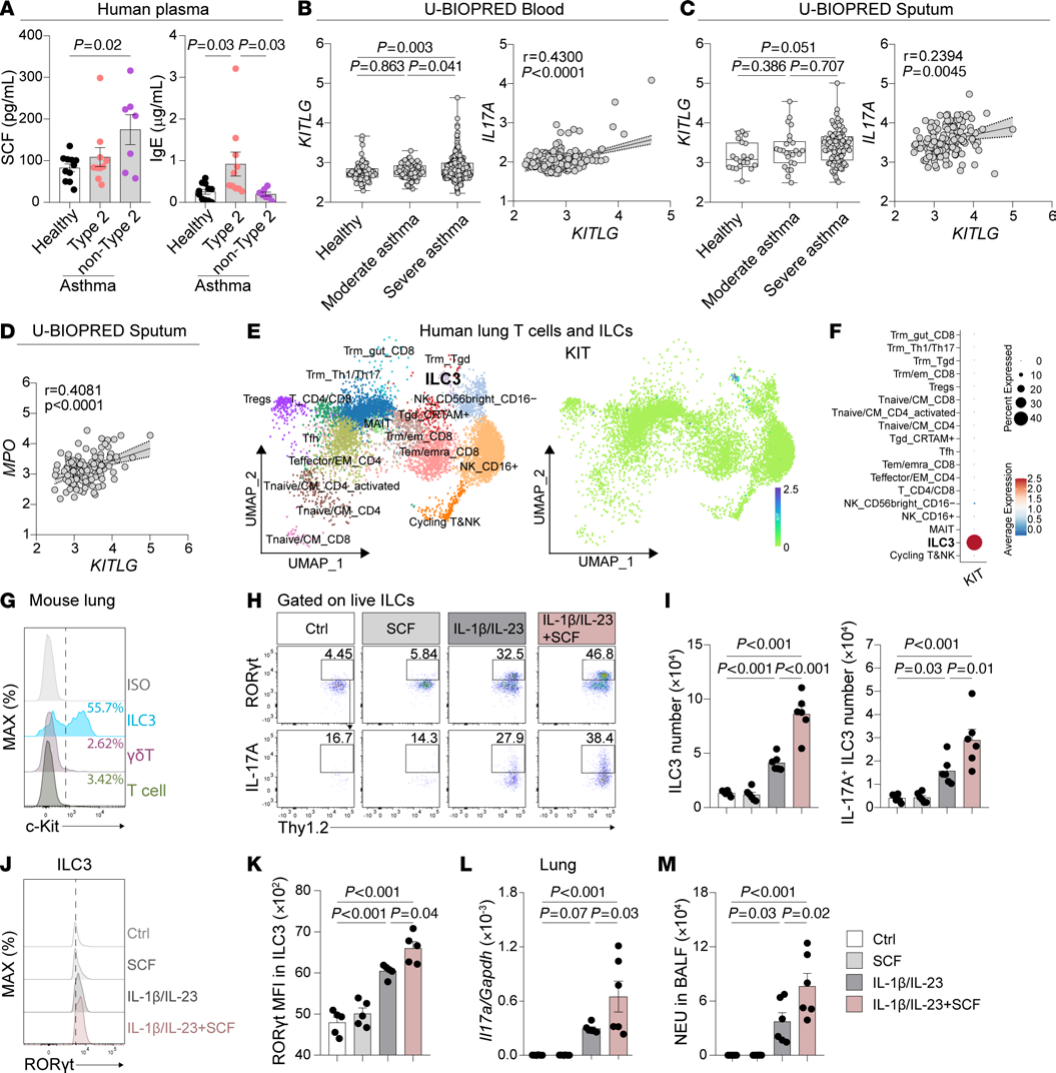
SCF expression correlates to IL-17A and MPO in asthmatic patients and synergizes with IL-1β/IL-23 to enhance ILC3 responses and neutrophilic inflammation in mouse model. (**A**) Protein levels of SCF and IgE in the plasma of Type 2 and non-Type 2 asthmatic patients and healthy controls. *n* = 7–11 per group. (**B**) U-BIOPRED blood gene expression for *KITLG* by severity cohort. Correlation between *KITLG* and *IL17A* gene expression in blood. (Pearson correlation, dotted line represents 95% CI) Data was obtained from GSE69683. *n* = 498 for healthy, moderate, and severe asthma. (**C** and **D**) U-BIOPRED induced sputum gene expression. Data was obtained from GSE76262. *n* = 139 for healthy, moderate, and severe asthma. (**C**) Gene expression for *KITLG* by severity cohort. Correlation between *KITLG* and *IL17A* gene expression in sputum. (Pearson correlation, dotted line represents 95% CI). (**D**) Correlation between *KITLG* and *MPO* gene expression in sputum. (Pearson correlation, dotted line represents 95% CI). (**E** and **F**) scRNA-seq of human lung T cells and ILCs. Data was obtained from the Human Cell Atlas. (**E**) Uniform Manifold Approximation and Projection (UMAP) plots of human lung T cells and ILCs and feature plots showing enrichment of KIT to cell clusters. (**F**) Dot plot for *KIT* expression of cell clusters. (**G**) Representative histograms of c-Kit expression in lung ILC3 (CD45^+^Thy1.2^+^Lin^-^ROR-γt^+^), γδT (CD45^+^Thy1.2^+^γδTCR^+^) and T cells (CD45^+^Thy1.2^+^Lin^+^γδTCR^-^) from C57BL/6 (WT) mice. (**H**–**M**) C57BL/6 (WT) mice intranasally (i.n.) received IL-1β/IL-23 and/or SCF for 3 days (Days 0–2) and were sacrificed on day 6 for following analysis. (**H**) Flow cytometry analysis of ILC3s. (**I**) Numbers of lung ILC3s (CD45^+^Thy1.2^+^Lin^-^ROR-γt^+^) and IL-17A^+^ ILC3s (CD45^+^Thy1.2^+^Lin^-^ROR-γt^+^IL-17A^+^). (**J**) Representative histograms of ROR-γt expression. (**K**) MFI of ROR-γt in lung ILC3s. (**L**) mRNA levels of *Il17a* in lung lysates. (**M**) Numbers of neutrophils (NEU) in BALF. *n* = 5–6 per group. Data are mean ± SEM and are representative of at least 2 independent experiments. Significance was determined by 1-way ANOVA (**A**–**C**, **I**, and **K**–**M**).

To determine SCF expression in asthmatic patients, we collected plasma samples from individuals with asthma and those who were healthy (referred to as healthy controls). We found that, unlike Type 2 asthmatic patients, non-Type 2 patients exhibited significantly higher plasma SCF protein levels compared with healthy controls (Figure 1A). Additionally, the elevated IgE expression in Type 2 asthmatic patients confirmed the presence of Type 2 inflammation (Figure 1A). These findings suggest that SCF is associated with non-Type 2 asthma, which is mainly mediated by neutrophilic inflammation (16, 49). To confirm this finding, we reanalyzed a published microarray dataset of peripheral blood and induced sputum samples from asthmatic participants in the Unbiased Biomarkers for the Prediction of Respiratory Disease Outcomes (U-BIOPRED) cohort (50, 51). Consistently, the reanalysis of blood samples demonstrated a significant upregulation of *KITLG* (the gene encoding SCF) expression in patients with severe asthma compared with those with moderate asthma and healthy controls (Figure 1B). More interestingly, we observed a positive correlation between *KITLG* and *IL17A* expression (Figure 1B). Similarly, *KITLG* expression was also higher in the sputum of patients with severe asthma compared with those with moderate asthma and healthy controls (Figure 1C). Moreover, in addition to its correlation with *IL17A* (Figure 1C), the expression of *KITLG* also positively correlated with the expression of *MPO*, a marker for neutrophils (Figure 1D). Given that IL-17A is associated with neutrophilic inflammation (28, 52), these findings suggest a participation of SCF in IL-17A production and neutrophilic inflammation during asthma.

### SCF enhances ILC3 activation and airway neutrophilia in response to IL-1β/IL-23.

We next investigated whether SCF could regulate IL-17A–producing cells. To identify the IL-17A–producing cells potentially regulated by SCF, we analyzed a previously published single cell RNA-seq (scRNAseq) dataset of T cells and innate lymphoid cells from human lung samples (53). Surprisingly, the reanalysis revealed that ILC3s are the major lymphocytes expressing *KIT,* the gene encoding SCF receptor c-Kit, in human lung (Figure 1E). Approximately 40% of ILC3s expressed *KIT*, whereas *KIT* expression in other lymphocyte populations was negligible (Figure 1F). To confirm this observation in mice, we performed flow cytometry to assess the expression of c-Kit on IL-17A–producing cells, including T cells, γδ-T cells, and ILC3s in the mouse lung. Consistently, while approximately half of the ILC3s expressed c-Kit, neither T cells nor γδ-T cells exhibited c-Kit expression on their surface in the mouse lung (Figure 1G). Based on these observations, we hypothesized that SCF might modulate lung ILC3 function. To test this, WT mice were intranasally (i.n.) treated with SCF. However, flow cytometric analysis of pulmonary ILC3s revealed that SCF alone did not alter the numbers of total ILC3s or IL-17A–producing ILC3s in the lung (Figure 1, H and I, and Supplemental Figure 1A; supplemental material available online with this article; https://doi.org/10.1172/JCI187372DS1). Since IL-1β/IL-23 are well known to activate ILC3, we then explored the potential cooperation between SCF and IL-1β/IL-23 in the activation of lung ILC3s. Interestingly, we found that the combination of IL-1β/IL-23 plus SCF led to increased numbers of ILC3s and IL-17A–producing ILC3s (Figure 1, H and I), compared with IL-1β/IL-23 alone, suggesting that SCF can enhance pulmonary ILC3 activation in response to IL-1β/IL-23. This enhanced ILC3 activation was accompanied by increased expression of ROR-γt, (Figure 1, J and K), which is associated with ILC3 function (54, 55). Consistent with the elevated ILC3 numbers, we observed increased *Il17a* mRNA levels in lung lysates of mice treated with the combination of IL-1β/IL-23 and SCF compared with those receiving IL-1β/IL-23 alone (Figure 1L). In humans, the epithelial IL-17A gene signature (56) and elevated IL-17A production in both serum and sputum (27, 52) were associated with airway neutrophilia. Consistent with this, the increase in IL-17A–producing ILC3s and *Il17a* mRNA levels were associated with the increased neutrophil counts in BALF (Figure 1M). These data suggest that SCF can work synergistically with IL-1β/IL-23 to enhance ILC3 activation and neutrophilic inflammation in the lung.

### SCF enhances ILC3 effector function and STAT3 pathway.

**Figure 2:**
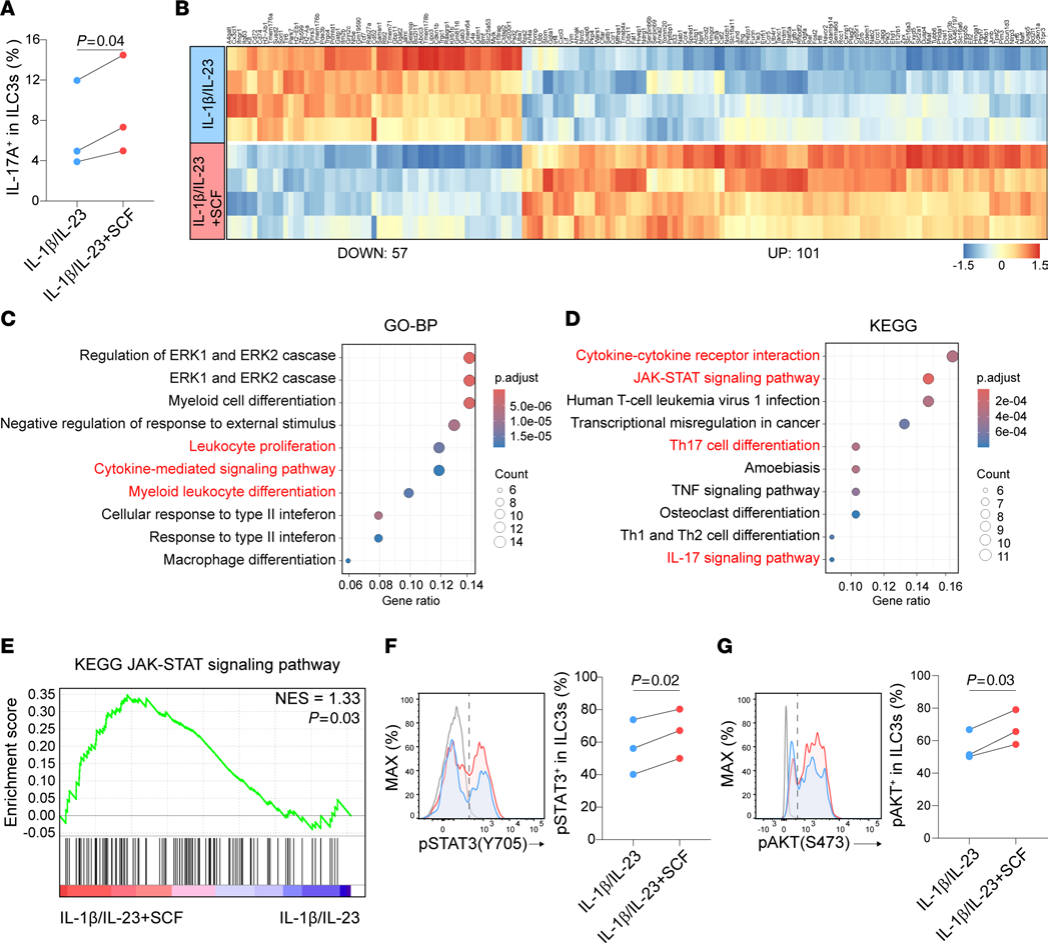
SCF promotes ILC3 effector function and STAT3 pathway. (**A**) Sorted lung ILCs (CD45^+^Thy1.2^+^Lin^-^) were treated with either IL-1β/IL-23 or IL-1β/IL-23/SCF for 48 hours and then analyzed by flow cytometry. Percentage of IL-17A**^+^** cells in ILC3s (CD45^+^Thy1.2^+^Lin^-^ROR-γt^+^). *n* = 3 per group. (**B**–**E**) Sorted small intestine ILC3s (CD45^int^Thy1.2^hi^Lin^-^KLRG1^–^) were treated with either IL-1β/IL-23 or IL-1β/IL-23/SCF for 6 hours and then subjected to RNA-seq analysis. (**B**) Heatmap showing differentially expressed genes, with a statistical cutoff of *P* < 0.005. (**C**) Gene Ontology (GO) biological process enrichment analyses of the genes upregulated in IL-1β/IL-23/SCF-treated ILC3s relative to IL-1β/IL-23 alone. (**D**) Kyoto Encyclopedia of Genes and Genomes (KEGG) enrichment analyses of the genes upregulated in IL-1β/IL-23/SCF-treated ILC3s relative to IL-1β/IL-23 alone. (**E**) Gene set enrichment analysis (GSEA) for KEGG JAK-STAT signaling pathway in IL-1β/IL-23/SCF-treated ILC3s relative to IL-1β/IL-23 alone. *n* = 4 per group. (**F** and **G**) Sorted lung ILCs (CD45^+^Thy1.2^+^Lin^-^) were treated with either IL-1β/IL-23 or IL-1β/IL-23/SCF for 10 minutes and then analyzed by flow cytometry. (**F**) Percentage of pSTAT3**^+^** cells in ILC3s (CD45^+^Thy1.2^+^Lin^-^ROR-γt^+^). *n* = 3 per group. (**G**) Percentage of pAKT^+^ cells in ILC3s. *n* = 3 per group. All sorted cells were pooled from at least 10 mice and the data are representative of at least 3 independent experiments. Significance was determined by 2-tailed paired Student’s *t* test (**A**, **F**, and **G**).

Since SCF increased the number of IL-17A–producing ILC3s in mice treated with IL-1β/IL-23, we next asked whether SCF can directly regulate IL-17A production from ILC3s. To test this, FACS-sorted lung ILCs were treated with either IL-1β/IL-23 or IL-1β/IL-23/SCF ex vivo. The flow cytometric analysis revealed that the addition of SCF could increase the frequency of IL-17A^+^ cells in ILC3s compared with those with IL-1β/IL-23 alone (Figure 2A). These data suggest that SCF can directly enhance ILC3 cytokine production in response to IL-1β/IL-23 stimulation.

To further investigate the genetic profile regulated by SCF treatment in an unbiased manner, we performed RNA-seq to analyze the transcriptome of ILC3s. ILC3s were stimulated with either IL-1β/IL-23 or IL-1β/IL-23 plus SCF ex vivo. We identified 158 differentially expressed genes between the two groups (Figure 2B). Notably, the genes upregulated in the IL-1β/IL-23 plus SCF group were involved in leukocyte proliferation, cytokine-mediated signaling pathway, and myeloid leukocyte differentiation by using Gene Ontology (GO) term enrichment analysis (Figure 2C). KEGG pathway analysis further highlighted the enrichment in cytokine-cytokine receptor interaction, Th17 cell differentiation, IL-17 signaling pathway, and JAK-STAT signaling pathway (Figure 2D), indicating enhanced effector function in ILC3s. Further, gene set enrichment analysis (GSEA) showed that the JAK-STAT signaling pathway was upregulated in the whole gene expression profiles from ILC3s treated with IL-1β/IL-23 plus SCF compared with IL-1β/IL-23 alone (Figure 2E), suggesting increased JAK-STAT signaling that may contribute to enhanced effector function of ILC3s. It is reported that STAT3 is critical for ROR-γt expression and ILC3 function (57–59). Therefore, we examined whether c-Kit engagement could regulate the phosphorylation of STAT3 in lung ILC3s by using phosphor-flow cytometry. Our data showed that the addition of SCF led to an enhancement in the percentage of phosphorylated STAT3 in ILC3s compared with IL-1β/IL-23 treatment alone (Figure 2F). Furthermore, given that the downstream of c-Kit includes PI3K/Akt (36), and AKT activation was shown to affect STAT3 phosphorylation in ILC3s (54), we then asked if c-Kit engagement enhanced AKT phosphorylation in lung ILC3s. Indeed, SCF treatment increased the phosphorylation of AKT in ILC3s treated with IL-1β/IL-23 plus SCF (Figure 2G). In summary, our data indicate that SCF/c-Kit signaling can directly alter the genetic profile and enhance the functions of ILC3s, which is associated with the enhanced AKT/STAT3/ROR-γt pathway.

### c-Kit signaling regulates IL-1β/IL-23–induced neutrophilic inflammation, AHR, and ILC3 activation.

Subsequently, to investigate the impact of c-Kit signaling on ILC3 function in vivo, we utilized WT and c-Kit–deficient (*Kit*^W-sh^) mice. Under steady state, no significant differences were observed in the numbers of lung ILC3s between WT and *Kit*^W-sh^ mice (Supplemental Figure 2A). Flow cytometric analysis also revealed similar levels of IL-17A secretion from ILC3s in both groups (Supplemental Figure 2B). We then asked whether c-Kit deficiency influenced the expression of other surface markers on ILC3s. Apart from the decreased expression of c-Kit on ILC3s in *Kit*^W-sh^ mice, other markers, such as Thy1.2, IL-23R, ICOS, Sca-1, CCR6,and NKp46, remained comparable between the 2 groups (Supplemental Figure 2B). These data indicate that c-Kit deficiency does not alter ILC3 function or the expression of other surface markers during the steady state condition.

**Figure 3:**
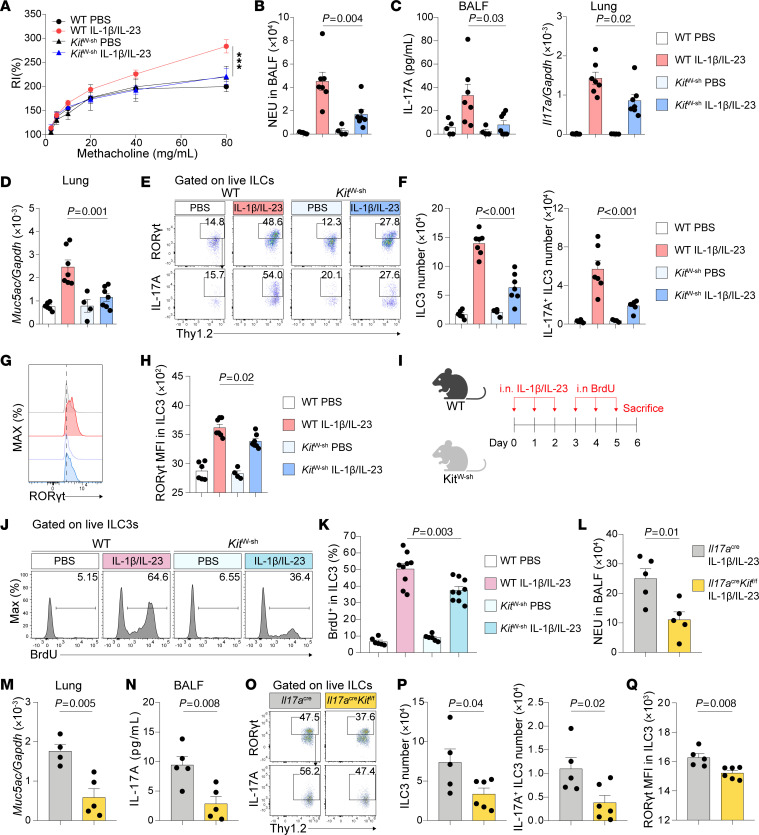
c-Kit deficiency in ILC3s ameliorates IL-1β/IL-23-induced neutrophilic inflammation, AHR, and ILC3 responses. (**A**–**H**) C57BL/6 (WT) and c-Kit deficient (*Kit*^W-sh^) mice intranasally (i.n.) received IL-1β and IL-23 for 3 days (Days 0–2) and were sacrificed on day 6 for analysis. (**A**) Lung resistance in response to increasing doses of methacholine. (**B**) Numbers of neutrophils (NEU) in BALF. (**C**) IL-17A protein levels in BALF and mRNA levels in lung lysates. (**D**) *Muc5ac* mRNA levels in lung lysates. (**E**) Flow cytometric analysis of ILC3s (**F**) Numbers of lung ILC3s (CD45^+^Thy1.2^+^Lin^-^ROR-γt^+^) and IL-17A^+^ ILC3s (CD45^+^Thy1.2^+^Lin^-^ROR-γt^+^IL-17A^+^). (**G**) Representative histograms of ROR-γt expression (**H**) MFI of ROR-γt in lung ILC3s. *n* = 4–7 per group. (**I**–**K**) C57BL/6 (WT) and c-Kit deficient (*Kit*^W-sh^) mice i.n. received IL-1β and IL-23 for 3 days (Days 0–2), BrdU for another 3 days (Days 3–5) and were sacrificed on day 6 for following analysis. (**I**) Experimental scheme. (**J**) Flow cytometry analysis of BrdU in ILC3s (CD45^+^Thy1.2^+^Lin^-^ROR-γt^+^). (**K**) Percentage of BrdU^+^ cells in ILC3s. *n* = 6–9 per group. (**L**–**Q**) *Il17a*^cre/+^ and *Il17a*^cre/+^*Kit*^fl/fl^ i.n. received IL-1β and IL-23 for 3 days (Days 0–2) and were sacrificed on day 6 for following analysis. (**L**) Numbers of NEU in BALF. (**M**) *Muc5ac* mRNA levels in lung lysates. (**N**) IL-17A protein levels in BALF. (**O**) Flow cytometric analysis of ILC3s (**P**) Numbers of lung ILC3s and IL-17A^+^ ILC3s. (**Q**) MFI of ROR-γt in lung ILC3s. *n* = 4–6 per group. Data are mean ± SEM and are representative of at least 2 independent experiments. Significance was determined by 2-way ANOVA (**A**), 1-way ANOVA (**B**–**D**, **F**, **H**, and **K**), and 2-tailed unpaired Student’s *t* test (**L**–**N** and **P**–**Q**); *P <.05; **P <.01; ***P <.001.

Next, to investigate the role of c-Kit signaling on IL-1β/IL-23–driven ILC3 activation and lung pathogenesis, we compared IL-1β/IL-23–induced neutrophilic inflammation, airway hyperreactivity (AHR), and ILC3 responses between c-Kit deficient (*Kit*^W-sh^) mice and their WT counterparts. Following intranasal IL-1β/IL-23 treatment, WT mice exhibited significantly increased AHR and neutrophil infiltration in the BALF compared with the controls (Figure 3, A and B). In contrast, *Kit*^W-sh^ mice demonstrated ameliorated airway resistance (Figure 3A) and reduced neutrophil infiltration (Figure 3B).

Previous studies have identified the important role of IL-17A in the development of AHR in both human and mouse models (24, 31). In line with these findings, our results demonstrated that IL-1β/IL-23–induced airway resistance in WT mice was accompanied by elevated IL-17A protein levels in BALF and increased *Il17a* mRNA expression in lung lysates (Figure 3C). Conversely, *Kit*^W-sh^ mice showed significantly lower IL-17A production at both protein and mRNA levels (Figure 3C). Furthermore, while IL-1β/IL-23 treatment induced the expression of *Muc5ac*, a mucus-associated gene, in lung lysates of WT mice, *Kit*^W-sh^ mice exhibited reduced *Muc5ac* expression (Figure 3D). Flow cytometric analysis revealed that IL-1β/IL-23 treatment increased the numbers of total lung ILC3s and IL-17A–producing ILC3s in WT mice, whereas *Kit*^W-sh^ mice exhibited reduced numbers of both ILC3s and IL-17A–producing ILC3s (Figure 3, E and F). Concomitantly, we observed a decrease in the expression of ROR-γt in lung ILC3s from *Kit*^W-sh^ mice compared with their WT counterparts (Figure 3, G and H). To assess whether c-Kit signaling regulates ILC3 proliferation, we employed intranasal BrdU administration (Figure 3I). Flow cytometric analysis revealed that, while IL-1β/IL-23 largely increased BrdU-labeled lung ILC3s in WT mice, c-Kit deficiency significantly reduced the frequency of BrdU-labeled ILC3s, indicating an impairment in ILC3 proliferation (Figure 3, J and K).

We next examined whether the inhibition of c-Kit signaling through the use of anti-c-Kit neutralizing antibody would yield similar outcomes as observed in c-Kit–deficient mice. IL-1β/IL-23–treated mice were intraperitoneally (i.p.) administered with anti–c-Kit neutralizing antibody (Supplemental Figure 3A), and the results showed that blocking c-Kit signaling ameliorated IL-1β/IL-23–induced airway resistance (Supplemental Figure 3B), decreased neutrophil infiltration (Supplemental Figure 3C),and reduced IL-17A production in BALF (Supplemental Figure 3D). Consistently, the numbers of ILC3s and IL-17A–producing ILC3s were also decreased following the treatment with anti–c-Kit neutralizing antibody, as compared with IgG controls (Supplemental Figure 3E). Taken together, these results suggest that the deficiency of c-Kit signaling reduced IL-1β/IL-23–induced neutrophilic inflammation, AHR, and ILC3 activation.

### ILC3-intrinsic c-Kit signaling supports IL-1β/IL-23–induced neutrophilic inflammation, AHR, and ILC3 activation.

To investigate the cell-intrinsic role of c-Kit signaling in ILC3, we generated *Il17a*^cre/+^*Kit*^fl/fl^ mice to delete *Kit* in IL-17A producing cells, a genetic engineering strategy used to target ILC3s (60). Since both lung T cells and γδ-T cells do not express c-Kit (Figure 1F), lung Th17 and γδT cells will not be targeted. Therefore, the effects of c-Kit knockout in ILC3s could be examined by comparing *Il17a*^cre/+^*Kit*^fl/fl^ mice with *Il17a*^cre/+^ control mice. Consistent with the observations from *Kit*^W-sh^ mice, we found ameliorated airway neutrophilia (Figure 3L), decreased mRNA levels of *Muc5ac* in lungs (Figure 3M), and reduced IL-17A production in BALF (Figure 3N) of *Il17a*^cre/+^*Kit*^fl/fl^ mice following IL-1β/IL-23 treatment compared with *Il17a*^cre/+^ control mice. Correspondingly, *Il17a*^cre/+^*Kit*^fl/fl^ mice exhibited lower numbers of ILC3s and IL-17A–producing ILC3s (Figure 3, O and P), and decreased MFI of ROR-γt in ILC3s (Figure 3Q) compared with those in *Il17a*^cre/+^ control mice. Notably, these results could be recapitulated in *Kit*^fl/fl^ crossed with *Il22*^cre/+^ mice, another mouse strain known to target ILC3s (60). When both *Il22*^cre/+^ mice and *IL22*^cre/+^*Kit*^fl/fl^ mice were treated with IL-1β/IL-23, *IL22*^cre/+^*Kit*^fl/fl^ mice also exhibited reduced neutrophil infiltration (Supplemental Figure 4A), lower BALF IL-17A production (Supplemental Figure 4B) and decreased numbers of lung ILC3s and IL-17A-producing ILC3s (Supplemental Figure 4C) compared with *Il22*^cre/+^ control mice. Collectively, these data indicate that the ILC3-intrinsic c-Kit signaling modulates ILC3 activation and neutrophilic inflammation.

### c-Kit signaling in ILC3s mediates LPS-induced neutrophilic airway inflammation, AHR, and ILC3 activation.

A previous study has shown that intranasal lipopolysaccharide (LPS) challenge induces neutrophilic inflammation and airway resistance in mice, which is associated with an increase in ILC3 numbers (34). In line with this, we observed that LPS challenge significantly induced AHR (Supplemental Figure 5A) and neutrophil infiltration in the BALF (Supplemental Figure 5B). Histological analysis of lung sections following LPS exposure revealed thickening of bronchiole epithelium and parenchymal cell infiltration, while Periodic acid–Schiff (PAS) staining showed increased mucus production in bronchial regions (Supplemental Figure 5C). Correspondingly, mRNA levels of mucus-associated gene *Muc5ac* were elevated in lung lysates after LPS exposure (Supplemental Figure 5D). Type 3–associated cytokines, including IL-1β, IL-23, IL-17A, and IL-22, were induced in BALF (Supplemental Figure 5E). Moreover, increased numbers of ILC3s and IL-17A–producing ILC3s were also observed after LPS challenge (Supplemental Figure 5F). Next, to assess the role of ILC3s in LPS-induced AHR and neutrophilic inflammation, we compared *Rag1*^–/–^ (which lack both T cells and B cells) and *Rag1*^–/–^*Rorc*^gfp/gfp^ mice (which lack T cells, B cells and ILC3s) with WT mice. Our data showed that both WT and *Rag1*^–/–^ mice exhibited comparable levels of airway resistance and neutrophil infiltration in the BALF (Supplemental Figure 5, G and H). However, *Rag1*^–/–^*Rorc*^gfp/gfp^ mice showed significantly reduced airway resistance and decreased neutrophil counts in BALF compared with those in WT and *Rag1*^–/–^ mice (Supplemental Figure 5, G and H). These suggest that ILC3s are sufficient for AHR and neutrophilia induced by LPS challenge. In addition, since IL-17A is associated with airway resistance and neutrophilic inflammation (24, 61, 62), we then ask whether IL-17A plays an important role in LPS-induced AHR by comparing *Il17a*^cre/cre^ mice (which are deficient in IL-17A) with WT mice. We found that the deficiency of IL-17A could ameliorate AHR (Supplemental Figure 5I) and neutrophilic inflammation (Supplemental Figure 5J) induced by LPS. As expected, no IL-17A could be detected in these mice upon LPS treatment (Supplemental Figure 5K). Together, these data indicated that ILC3s can mediate LPS-induced AHR and neutrophilic inflammation through IL-17A production.

**Figure 4:**
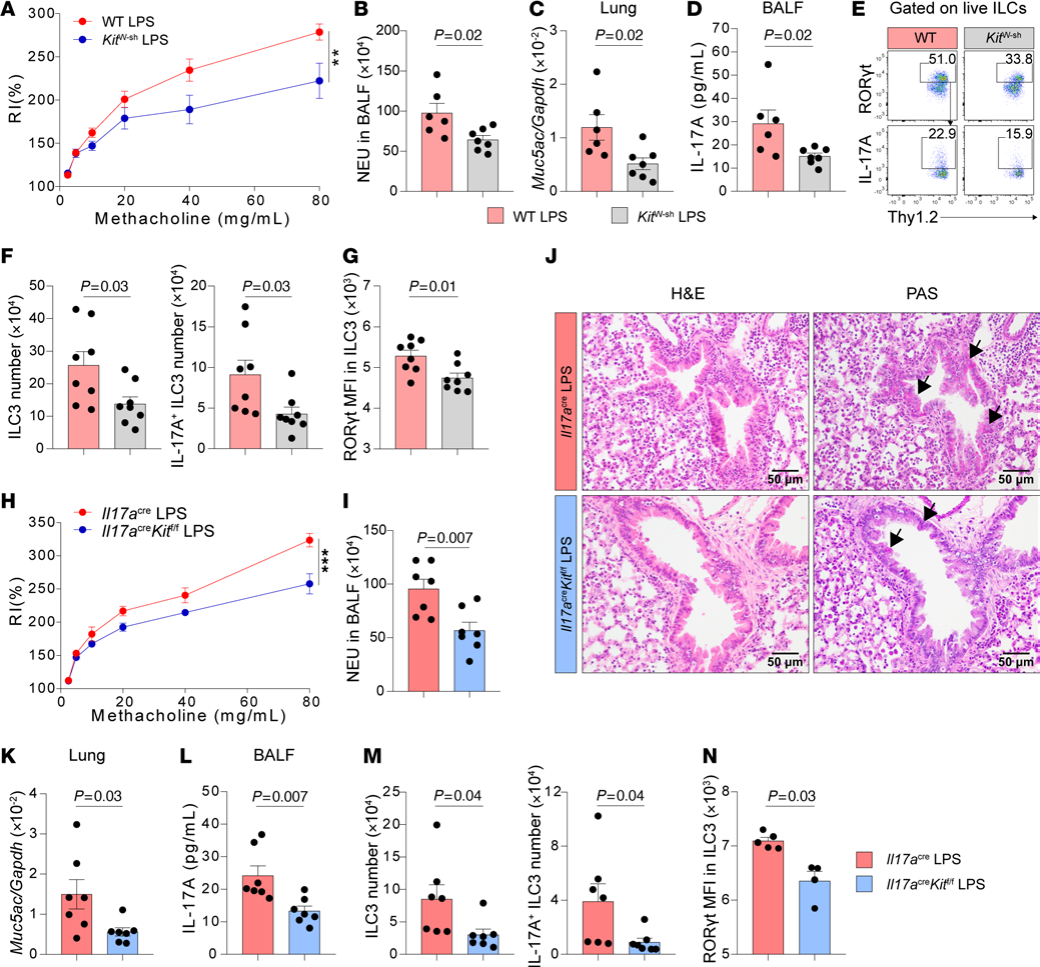
c-Kit deficiency in ILC3s alleviates LPS-induced neutrophilic inflammation, AHR, and ILC3 responses. (**A**–**G**) C57BL/6 (WT) and c-Kit deficient (*Kit*^W-sh^) mice intranasally (i.n.) received LPS for 4 days (Days 0–3) and were sacrificed on day 4 for analysis. (**A**) Lung resistance in response to increasing doses of methacholine. (**B**) Numbers of neutrophils (NEU) in BALF. (**C**) *Muc5ac* mRNA levels in lung lysates. (**D**) IL-17A protein levels in BALF. (**E**) Flow cytometry analysis of ILC3s. (**F**) Numbers of lung ILC3s (CD45^+^Thy1.2^+^Lin^-^ROR-γt^+^) and IL-17A^+^ ILC3s (CD45^+^Thy1.2^+^Lin^-^ROR-γt^+^IL-17A^+^). (**G**) MFI of ROR-γt in lung ILC3s. *n* = 6–8 per group. (**H**–**N**) *Il17a*^cre/+^ and *Il17a*^cre/+^*Kit*^fl/fl^ i.n. received LPS for 4 days (Days 0–3) and were sacrificed on day 4 for analysis. (**H**) Lung resistance in response to increasing doses of methacholine. (**I**) Numbers of NEU in BALF. (**J**) Representative histological images of H&E and Periodic acid–Schiff (PAS) staining of the lung tissue. The arrows indicated the mucus deposition. Scale bars: 50μm. (**K**) *Muc5ac* mRNA levels in lung lysates. (**L**) IL-17A protein levels in BALF. (**M**) Numbers of lung ILC3s and IL-17A^+^ ILC3s. (**N**) MFI of ROR-γt in lung ILC3s. *n* = 4–7 per group. Data are mean ± SEM and are representative of at least 2 independent experiments. Significance was determined by 2-way ANOVA (**A** and **H**) and 2-tailed unpaired Student’s *t* test (**B**–**D**, **F**, **G**, **I**, and **K**–**N**); *P <.05; **P <.01; ***P <.001.

We then explored the impact of c-Kit signaling in LPS-induced ILC3 activation and lung pathogenesis by comparing WT and *Kit*^W-sh^ mice. The results showed that, upon LPS exposure, *Kit*^W-sh^ mice exhibited ameliorated AHR (Figure 4A), reduced BALF neutrophilia (Figure 4B), and lower *Muc5ac* mRNA levels (Figure 4C) compared with controls. Furthermore, the protein level of IL-17A was reduced in *Kit*^W-sh^ mice (Figure 4D). Notably, the reduction in inflammation was associated with the decrease in the numbers of ILC3s and IL-17A–producing ILC3s (Figure 4, E and F). Moreover, we also observed a lower MFI of ROR-γt in ILC3s of *Kit*^W-sh^ mice compared with WT mice (Figure 4G).

To further confirm the role of c-Kit signaling in ILC3s, we employed *Il17a*^cre/+^*Kit*^fl/fl^ mice in this LPS-induced neutrophilic asthma model. Consistent with our previous findings, *Il17a*^cre/+^*Kit*^fl/fl^ mice exhibited lower airway resistance (Figure 4H) and reduced neutrophil infiltration (Figure 4I), compared with *Il17a*^cre/+^ control mice. Histological examination of lung sections revealed decreased airway inflammation, and PAS staining demonstrated reduced mucus production in the *Il17a*^cre/+^*Kit*^fl/fl^ mice (Figure 4J). Accordingly, qPCR analysis showed lower mRNA levels of *Muc5ac* in lung lysates of *Il17a*^cre/+^*Kit*^fl/fl^ mice (Figure 4K). Moreover, we observed lower protein levels of IL-17A in BALF (Figure 4L) and decreased numbers of lung ILC3s and IL-17A–producing ILC3s (Figure 4M) of *Il17a*^cre/+^*Kit*^fl/fl^ mice compared with *Il17a*^cre/+^ controls. The MFI of ROR-γt in ILC3s was also lower in *Il17a*^cre/+^*Kit*^fl/fl^ mice (Figure 4N). Additional experiments using *Il22*^cre/+^*Kit*^fl/fl^ mice further supported these findings, demonstrating reduced neutrophilic inflammation, decreased *Il17a* and *Muc5ac* expression, and lower numbers of ILC3s and IL-17A–producing ILC3s upon LPS challenge (Supplemental Figure 4, D–F). *Rorc*^cre^*Kit*^fl/fl^ mice also exhibited reduced neutrophil infiltration (Supplemental Figure 4G), lower IL-17A protein levels in BALF (Supplemental Figure 4H), and decreased numbers of ILC3s and IL-17A–producing ILC3s in lung (Supplemental Figure 4I). Collectively, these data suggest that the lack of c-Kit signaling in ILC3s could alleviate ILC3 activation and the severity of neutrophilic inflammation upon LPS challenge.

### Fibroblast-derived SCF supports ILC3 activation and neutrophilic inflammation.

**Figure 5:**
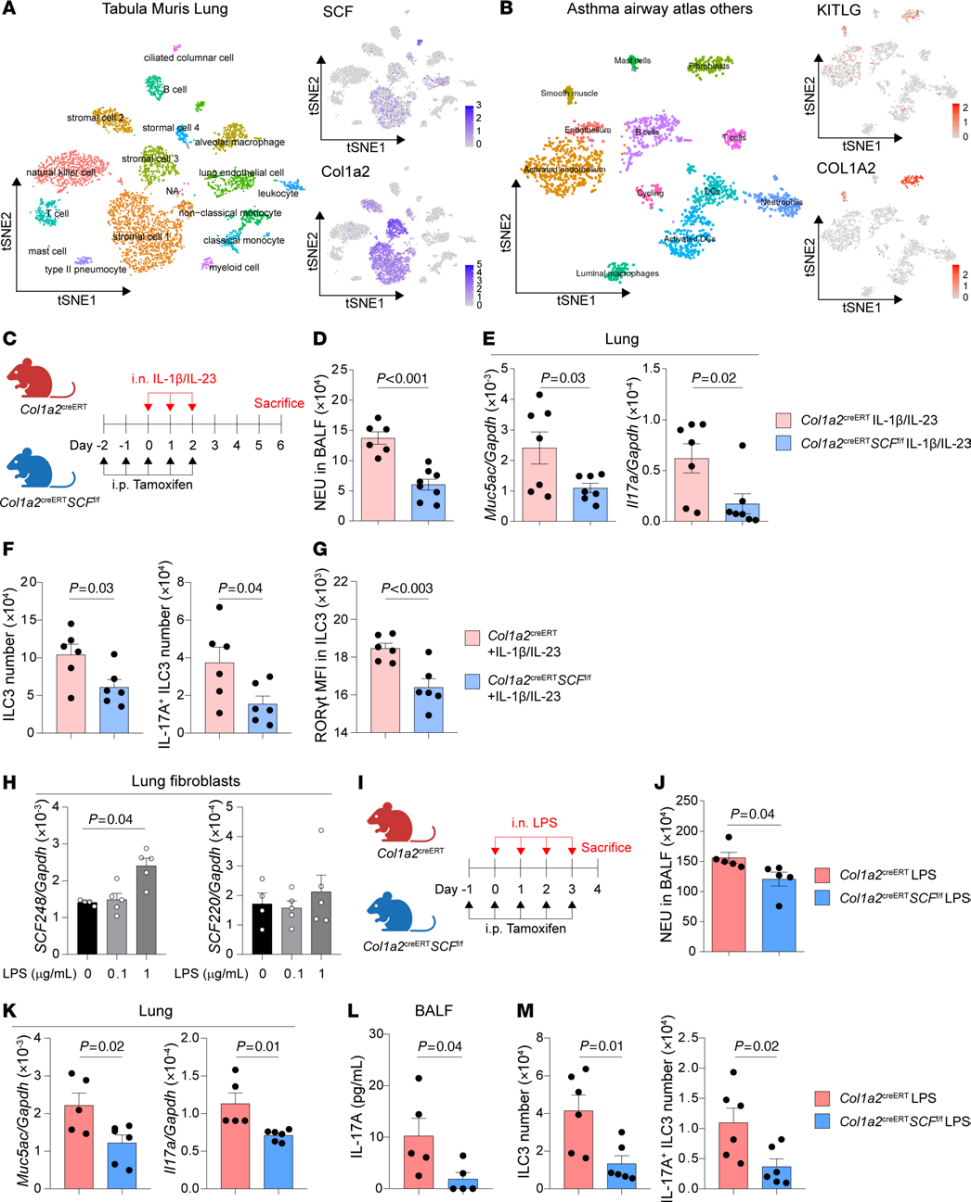
Conditional deletion of SCF in fibroblasts suppresses ILC3 responses and neutrophilic inflammation. (**A**) T-distributed stochastic neighbor embedding (t-SNE) plots of mouse lung cells and feature plots showing enrichment of *SCF* and *Col1a2* to cell clusters. (Data obtained from the Gene Expression Omnibus under accession no. GSE109774). (**B**) T-SNE plots of human asthmatic lung cells and feature plots showing enrichment of *KITLG* (*SCF*) and *COL1A2* to cell clusters. (Data obtained from the Human Cell Atlas [ref. 64]). (**C**–**G**) *Col1a2*^creERT^ and *Col1a2*^creERT^*SCF*^fl/fl^ mice i.p. received tamoxifen for 5 days (Day –2 to 2), i.n. received IL-1β and IL-23 for 3 days (Days 0–2) and were sacrificed on day 6 for analysis. (**C**) Experimental scheme. (**D**) Numbers of neutrophils (NEU) in BALF. (**E**) *Muc5ac* and *Il17*a mRNA levels in lung lysates. (**F**) Numbers of lung ILC3s (CD45^+^Thy1.2^+^Lin^-^ROR-γt^+^) and IL-17A^+^ ILC3s (CD45^+^Thy1.2^+^Lin^-^ROR-γt^+^IL-17A^+^). (**G**) MFI of ROR-γt in lung ILC3s. *n* = 6–8 per group. (**H**) mRNA levels of *SCF248* and *SCF220* on isolated pulmonary fibroblasts treated with different dose of LPS for 24 hours. *n* = 4–5 per group. (**I**–**M**) *Col1a2*^creERT^ and *Col1a2*^creERT^*SCF*^fl/fl^ mice i.p. received tamoxifen for 5 days (Days –1–3), i.n. received LPS for 4 days (Days 0–3) and were sacrificed on day 4 for analysis. (**I**) Experimental scheme. (**J**) Numbers of NEU in BALF. (**K**) *Muc5ac* and *Il17*a mRNA levels in lung lysates. (**L**) IL-17A protein levels in BALF. (**M**) Numbers of lung ILC3s and IL-17A^+^ ILC3s. *n* = 5–6 per group. Data are mean ± SEM and are representative of at least 2 independent experiments. Significance was determined by 2-tailed unpaired Student’s *t* test (**D**–**G** and **J**–**M**) and 1-way ANOVA (**H**).KITLG, KIT ligand.

To identify pulmonary cellular source of the c-Kit ligand, SCF, we analyzed a previously published scRNA-seq dataset of mouse whole lung cells to uncover SCF-secreting cells in the lung (63). The reanalysis revealed that SCF was primarily expressed in stromal cells expressing *Col1a2*, a fibroblast marker, implicating fibroblasts as potential SCF sources in mouse lung (Figure 5A). Moreover, to confirm this finding in asthmatic patients, we analyzed the scRNA-seq dataset of asthmatic lungs (64). The reanalysis also revealed the coexpression of *SCF* (*KITLG*) and *COL1A2* in human lung fibroblasts (Figure 5B), confirming the expression of SCF in human fibroblasts during asthma. Therefore, to examine the importance of SCF production by fibroblasts in the mouse model, we generated mice with inducible, fibroblast-specific SCF deletion (*Col1a2*^CreERT^*SCF*^fl/fl^ mice). Tamoxifen was i.p. administered in *Col1a2*^CreERT^*SCF*^fl/fl^ mice 2 days before IL-1β/IL-23 treatment for 5 consecutive days, and tamoxifen-treated *Col1a2*^CreERT^ mice were used as controls (Figure 5C). The data showed that following IL-1β/IL-23 treatment, SCF knockout in fibroblasts reduced airway neutrophilia (Figure 5D) and downregulated mRNA levels of *Muc5ac* and *Il17a* in lung lysates (Figure 5E). The number of ILC3s and IL-17A–producing ILC3s in lungs were decreased in *Col1a2*^CreERT^*SCF*^fl/fl^ mice (Figure 5F), compared with *Col1a2*^CreER^ mice. Moreover, a lower MFI of ROR-γt in ILC3s was also observed in mice with SCF knockout in fibroblasts (Figure 5G).

To investigate whether LPS triggered SCF expression in pulmonary fibroblasts, we examined the mRNA levels of 2 isoforms of SCF, SCF248 and SCF220, in lung primary fibroblasts. Our analysis revealed a dose-dependent increase in SCF248 mRNA levels after LPS exposure, whereas SCF220 mRNA levels remained unchanged in the lung fibroblasts (Figure 5H). These data suggest that LPS mainly induces the expression of soluble SCF (SCF248) from lung fibroblasts. Moreover, to examine the role of fibroblast-derived SCF in LPS-triggered ILC3 responses and neutrophilic inflammation, *Col1a2*^CreERT^*SCF*^fl/fl^ mice were treated with LPS (Figure 5I). *Col1a2*^CreERT^*SCF*^fl/fl^ mice also exhibited lower neutrophilic inflammation (Figure 5J), downregulated mRNA levels of *Muc5ac* and *Il17a* (Figure 5K), lower IL-17A production (Figure 5L), and decreased numbers of ILC3s and IL-17A–producing ILC3s (Figure 5M), compared with *Col1a2*^CreER^ control mice. These results suggest that fibroblasts are a major source of SCF for ILC3 activation in both IL-1β/IL-23- and LPS-induced neutrophilic inflammation, highlighting the importance of the fibroblast-ILC3 axis in the pathogenesis of neutrophilic inflammation.

### c-Kit signaling in ILC3s modulates PM_2.5_-induced neutrophilic inflammation, AHR, and ILC3 activation.

**Figure 6:**
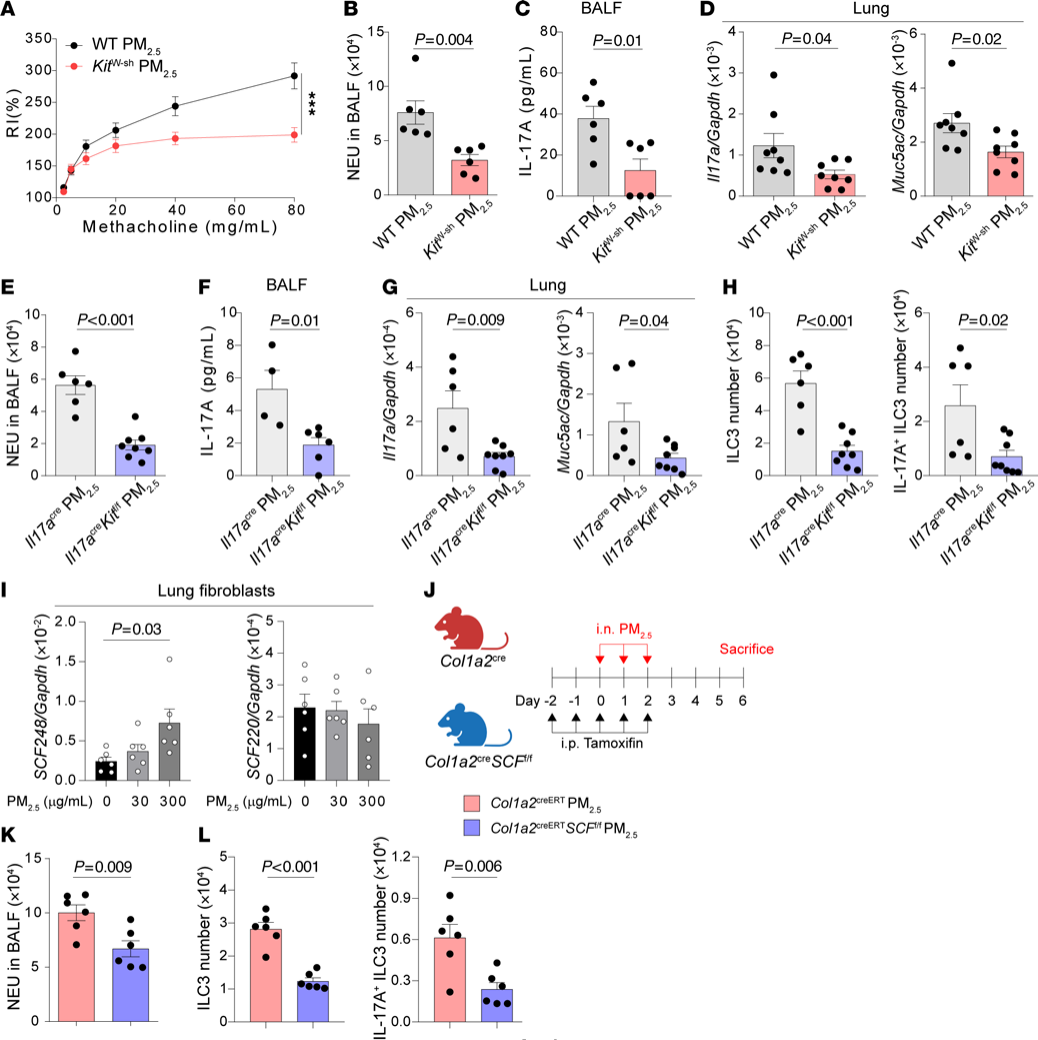
c-Kit deficiency suppresses ILC3 responses in PM_2.5_-induced neutrophilic inflammation. (**A**–**D**) C57BL/6 (WT) and c-Kit deficient (*Kit*^W-sh^) mice intranasally (i.n.) received PM_2.5_ for 3 days (Days 0–2) and were sacrificed on day 6 for analysis. (**A**) Lung resistance in response to increasing doses of methacholine. (**B**) Numbers of neutrophils (NEU) in BALF. (**C**) IL-17A protein levels in BALF. (**D**) *Muc5ac* and *Il17*a mRNA levels in lung lysates. *n* = 6–8 per group. (**E**–**H**) *Il17a*^cre/+^ and *Il17a*^cre/+^*Kit*^fl/fl^ mice i.n. received PM_2.5_ for 3 days (Days 0–2) and were sacrificed on day 6 for following analysis. (**E**) Numbers of NEU in BALF. (**F**) IL-17A protein levels in BALF. (**G**) *Il17a* and *Muc5ac* mRNA levels in lung lysates. (**H**) Numbers of lung ILC3s (CD45^+^Thy1.2^+^Lin^-^ROR-γt^+^) and IL-17A^+^ ILC3s (CD45^+^Thy1.2^+^Lin^-^ROR-γt^+^IL-17A^+^). *n* = 4–8 per group. (**I**) mRNA levels of *SCF248* and *SCF220* on isolated pulmonary fibroblasts treated with different dose of PM_2.5_ for 24 hours. *n* = 6 per group. (**J**–**L**) *Col1a2*^creERT^ and *Col1a2*^creERT^*SCF*^fl/fl^ mice i.p. received tamoxifen for 5 days (Days –2–2), i.n. received PM_2.5_ for 3 days (Days 0–2) and were sacrificed on day 6 for analysis. (**J**) Experimental scheme. (**K**) Numbers of NEU in BALF. (**L**) Numbers of lung ILC3s and IL-17A^+^ ILC3s. *n* = 6 per group. Data are mean ± SEM and are representative of at least 2 independent experiments. Significance was determined by 2-way ANOVA (**A**), 2-tailed unpaired Student’s *t* test (**B**–**H**, **K**, and **L**) and 1-way ANOVA (**I**), and; *P <.05; **P <.01; ***P <.001.

Given that our previous study has demonstrated that PM_2.5_ induces neutrophilic inflammation and ILC3 activation in the lung (65), we then asked whether c-Kit deficiency affected PM_2.5_-induced neutrophilic inflammation and ILC3 activation. To test this, WT and *Kit*^w-sh^ mice were challenged with PM_2.5_. Consistent with our previous observations, *Kit*^w-sh^ mice exhibited reduced airway resistance (Figure 6A), lower neutrophilia (Figure 6B), and decreased protein levels of IL-17A in BALF (Figure 6C). Additionally, we detected lower mRNA levels of *Il17a* and *Muc5ac* in *Kit*^w-sh^ mice (Figure 6D), compared with WT mice. Moreover, when *Il17a*^cre/+^*Kit*^fl/fl^ mice was used in the PM_2.5_ model, *Il17a*^cre/+^*Kit*^fl/fl^ mice also exhibited lower neutrophilia (Figure 6E) and IL-17A protein level in BALF (Figure 6F). We also detected decreases in mRNA levels of *Il17a* and *Muc5ac* in lung lysates of *Il17a*^cre/+^*Kit*^fl/fl^ mice (Figure 6G). Consistently, Flow cytometric analysis revealed that the numbers of ILC3s and IL-17A–producing ILC3s were reduced in *Il17a*^cre/+^*Kit*^fl/fl^ mice (Figure 6H) compared with the controls. Surprisingly, similar to LPS, we found that PM_2.5_ induced the mRNA level of SCF248, but not SCF220, in pulmonary fibroblasts (Figure 6I). By utilizing the *Col1a2*^CreERT^*SCF*^fl/fl^ mice (Figure 6J), we also detected lower neutrophilia (Figure 6K) and decreased ILC3s and IL-17A–producing ILC3s numbers (Figure 6L). These results also suggest an important role of SCF/c-Kit signaling in PM_2.5_-induced ILC3 activation and neutrophilic inflammation.

### Imatinib ameliorates neutrophilic inflammation, AHR, and ILC3 activation.

**Figure 7:**
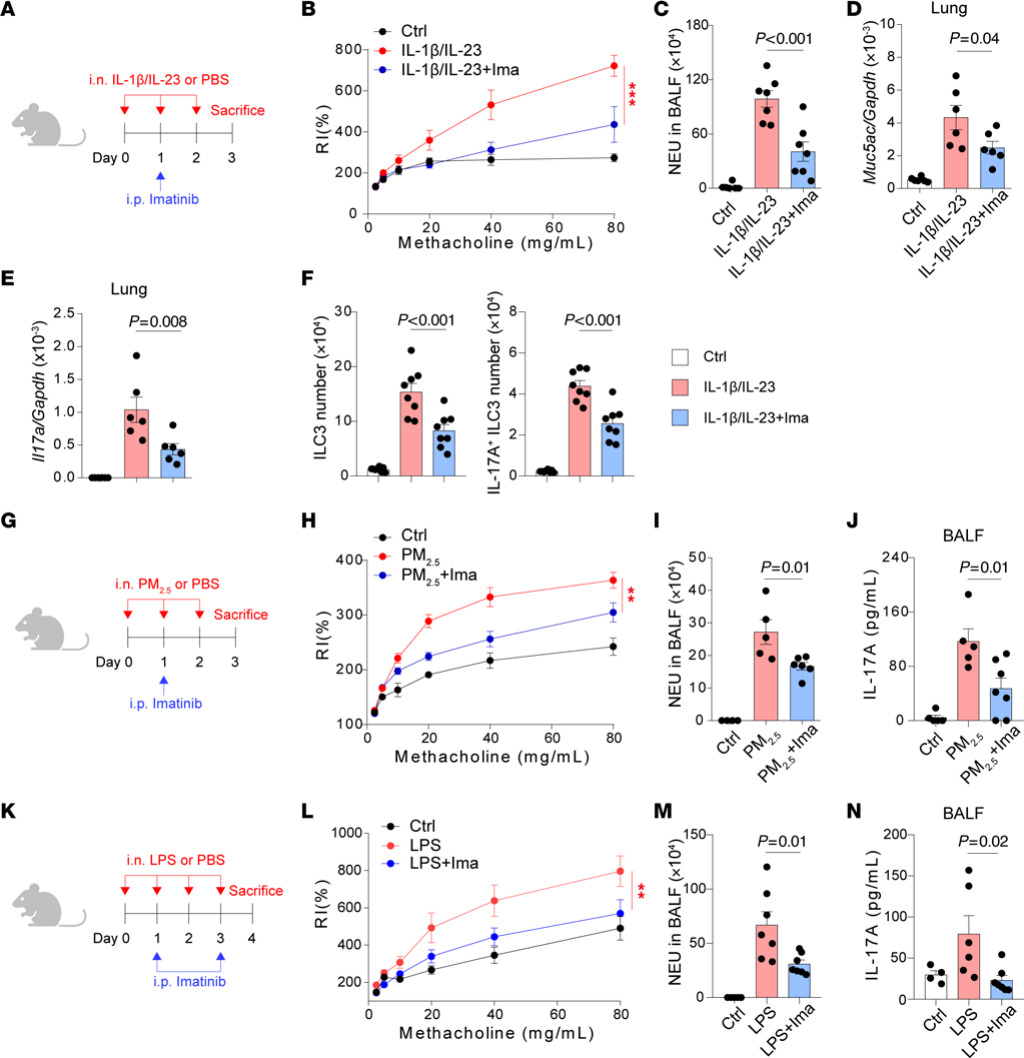
c-Kit inhibition by imatinib ameliorates neutrophilic inflammation, AHR, and ILC3 responses. (**A**–**F**) Balb/c (WT) intranasally (i.n.) received IL-1β and IL-23 for 3 days (Days 0–2), i.p. received imatinib on day 1 and were sacrificed on day 3 for following analysis. (**A**) Experimental scheme. (**B**) Lung resistance in response to increasing doses of methacholine. (**C**) Numbers of neutrophils (NEU) in BALF. (**D**) *Muc5ac* mRNA levels in lung lysates. (**E**) *Il17*a mRNA levels in lung lysates. (**F**) Numbers of lung ILC3s (CD45^+^Thy1.2^+^Lin^-^ROR-γt^+^) and IL-17A^+^ ILC3s (CD45^+^Thy1.2^+^Lin^-^ROR-γt^+^IL-17A^+^). *n* = 6–8 per group. (**G**–**J**) Balb/c (WT) i.n. received PM_2.5_ for 3 days (Days 0–2), i.p. received imatinib on day 1 and were sacrificed on day 3 for analysis. (**G**) Experimental scheme. (**H**) Lung resistance in response to increasing doses of methacholine. (**I**) Numbers of NEU in BALF. (**J**) IL-17A protein levels in BALF. *n* = 4–7 per group. (**K**–**N**) Balb/c (WT) i.n. received LPS for 4 days (Days 0–3), i.p. received imatinib on day 1 and day 3, and were sacrificed on day 4 for analysis. (**K**) Experimental scheme. (**L**) Lung resistance in response to increasing doses of methacholine. (**M**) Numbers of NEU in BALF. (**N**) IL-17A protein levels in BALF. *n* = 4–9 per group. Data are mean ± SEM and are representative of at least 2 independent experiments. Significance was determined by 2-way ANOVA (**B**, **H**, and **L**) and 1-way ANOVA (**C**–**F**, **I**, **J** **M**, and **N**); *P <.05; **P <.01; ***P <.001.

To examine the therapeutic potential of targeting c-Kit signaling in neutrophilic asthma, Imatinib, a c-Kit inhibitor, was used to treat IL-1β/IL-23–induced neutrophilic asthma model (Figure 7A). The results showed that the treatment of imatinib could ameliorate AHR (Figure 7B) and neutrophil infiltration in BALF (Figure 7C). The levels of *Muc5ac* and *Il17a* mRNA were decreased when treated with imatinib (Figure 7, D and E). The numbers of ILC3s and IL-17A–producing ILC3s were also decreased by imatinib treatment (Figure 7F). To exclude the effects of T cells, we then asked whether imatinib could reduce ILC3 activation in the absence of adaptive immunity. *Rag*2^–/–^ mice (which lack T cells and B cells) were i.n. treated with IL-1β/IL-23 and i.p. administered with imatinib (Supplemental Figure 6A). The data showed that imatinib treatment significantly reduced lung ILC3 and IL-17A–producing ILC3 numbers (Supplemental Figure 6B) and decreased IL-17A production in BALF (Supplemental Figure 6C), showing that imatinib could reduce ILC3 activation independently of acquired immunity. These data revealed that imatinib alleviates ILC3 activation and neutrophilic inflammation in the presence and absence of adaptive immunity. Additionally, we further extended our investigation to PM_2.5_ and LPS-induced mouse neutrophilic asthma models (Figure 7, G and K). We found that imatinib treatment resulted in ameliorated AHR (Figure 7, H and L), reduced neutrophilia (Figure 7, I and M), and lower BALF IL-17A production (Figure 7, J and N) in these 2 mouse neutrophilic asthma models. Collectively, these results suggest a therapeutic potential of targeting SCF/c-Kit signaling by using imatinib in neutrophilic asthma.

To evaluate the involvement of mast cells in our models, we assessed lung mast cell numbers following treatment with IL-1β/IL-23 or LPS and used influenza infection as a positive control for mast cell induction (66). Toluidine blue staining was employed to identify mast cells and mast cell degranulation. We found that influenza infection significantly increased the numbers and degranulation of mast cells in the lungs, and both IL-1β/IL-23 and LPS stimulation also led to an increase in mast cell numbers and degranulation (Supplemental Figure 7, A and B). Furthermore, imatinib treatment did not alter mast cell numbers or their degranulation in the IL-1β/IL-23 model (Supplemental Figure 7, C–E). To further investigate the role of mast cells in the development of AHR, we adoptively transferred WT bone marrow–derived mast cells (BMMCs) into *Kit*^W-sh^ mice (Supplemental Figure 7F). The results showed that *Kit*^W-sh^ mice lacked detectable mast cells in the lungs. While the transfer of BMMCs could reconstitute mast cell populations in the lungs of *Kit*^W-sh^ mice, it had no effect on AHR and neutrophil numbers in BALF (Supplemental Figure 7, G and I–K). Following IL-1β/IL-23 stimulation, *Kit*^W-sh^ mice continued to exhibit a deficiency in lung mast cells, attenuated AHR, and decreased neutrophil counts in BALF compared to their WT counterparts (Supplemental Figure 7, H–K). Although the transfer of BMMCs restored the numbers of mast cells in IL-1β/IL-23–treated *Kit*^W-sh^ mice, it failed to rescue AHR or elevate BALF neutrophil counts to the levels observed in IL-1β/IL-23–treated WT mice (Supplemental Figure 7, H–K). These findings suggest that mast cells play a limited role in our models.

### c-Kit signaling promotes OVA/LPS-induced neutrophilic airway inflammation, AHR, and ILC3 activation.

**Figure 8:**
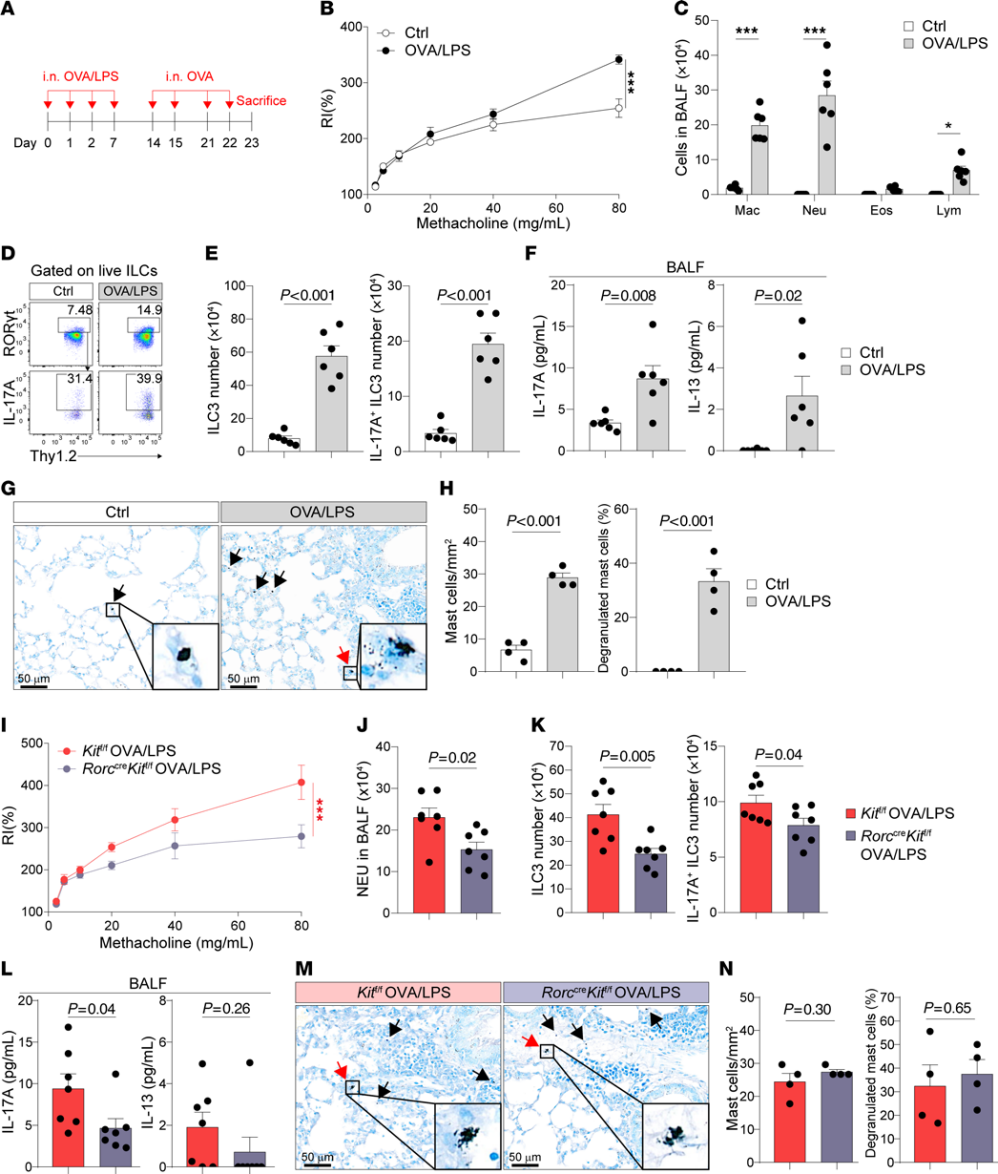
c-Kit deficiency in ILC3s mediates OVA/LPS-induced neutrophilic inflammation, AHR, and ILC3 responses. (**A**–**H**) C57BL/6 (WT) mice were i.n. administered OVA/LPS on days 0, 1, 2, and 7, followed by OVA on days 14, 15, 21, and 22. Mice were sacrificed on day 23 for analysis. (**A**) Experimental scheme. (**B**) Lung resistance in response to increasing doses of methacholine. (**C**) Cellular composition in BALF. Mac, macrophage; Neu, neutrophil; Eos, eosinophil; Lym, lymphocyte. (**D**) Flow cytometry analysis of ILC3s. (**E**) Numbers of lung ILC3s (CD45^+^Thy1.2^+^Lin^-^ROR-γt^+^) and IL-17A^+^ ILC3s (CD45^+^Thy1.2^+^Lin^-^ROR-γt^+^IL-17A^+^). (**F**) IL-17A and IL-13 protein levels in BALF. (**G**) Representative images of toluidine blue–stained lung sections. (**H**) Numbers of mast cells and percentage of degranulated mast cells in lungs. *n* = 4–6 per group. (**I**–**N**) *Kit*^fl/fl^ and *Rorc*^cre^*Kit*^fl/fl^ mice i.n. received OVA/LPS on days 0, 1, 2, and 7, and OVA on day 14, 15, 21, and 22. Mice were sacrificed on day 23 for subsequent analysis. (**I**) Lung resistance in response to increasing doses of methacholine. (**J**) Numbers of NEU in BALF. (**K**) Numbers of lung ILC3s and IL-17A^+^ ILC3s. (**L**) IL-17A and IL-13 protein levels in BALF. (**M**) Representative images of toluidine blue–stained lung sections. (**N**) Numbers of mast cells and percentage of degranulated mast cells in lungs. *n* = 4–7 per group. Data are means ± SEM and are representative of at least 2 independent experiments. For **G** and **M**, black arrows indicated mast cells and red arrows indicated degranulated mast cells. Scale bars: 50 μm. Significance was determined by 2-way ANOVA (**B** and **I**), multiple 2-tailed *t* test (**C**), and 2-tailed unpaired Student’s *t* test (**E**, **F**, **H**, **J**–**L**, and **N**); *P <.05; **P <.01; ***P <.001.

The ovalbumin (OVA)/LPS-driven mouse model is a well-established chronic experimental model for neutrophilic asthma, which is also associated with ILC3 activation (Figure 8A) (35, 67). Our data demonstrated that OVA/LPS exposure induced AHR, neutrophil infiltration, and increased numbers of lung ILC3s and IL-17A–producing ILC3s (Figure 8, B–E). IL-17A and IL-13 were elevated in BALF following OVA/LPS treatment (Figure 8F). Toluidine blue staining also revealed increased mast cell accumulation and degranulation in the lungs (Figure 8, G and H). To assess the role of c-Kit signaling in ILC3 within the OVA/LPS model, *Rorc*^cre^*Kit*^fl/fl^ mice were used. Compared with *Kit*^fl/fl^ control mice, OVA/LPS-treated *Rorc*^cre^*Kit*^fl/fl^ mice exhibited reduced AHR, decreased neutrophil infiltration, and lower numbers of ILC3s and IL-17A–producing ILC3s (Figure 8, I–K). The levels of IL-17A and IL-13 in BALF were reduced although the changes in IL-13 did not reach significant (Figure 8L). In addition, mast cell numbers and their degranulation remained unchanged in *Rorc*^cre^*Kit*^fl/fl^ mice compared with *Kit*^fl/fl^ control mice (Figure 8, M and N). Furthermore, imatinib treatment also led to a reduction in AHR and neutrophil infiltration, accompanied by decreased numbers of ILC3s, IL-17A–producing ILC3s, and reduced mast cell accumulation and degranulation (Supplemental Figure 8, A–F). Imatinib treatment also significantly reduced BALF IL-17A levels but not IL-13 (Supplemental Figure 8G). Collectively, these data suggest that c-Kit signaling in ILC3s plays a role in mediating OVA/LPS-induced neutrophilic inflammation.

Taken together, our study revealed a significant upregulation of SCF expression in patients with asthma, which correlated with the expressions of IL-17A and MPO. Importantly, we demonstrated that fibroblast-derived SCF is critical for optimal ILC3 activation and IL-17A production, which is associated with enhanced AKT/STAT3/ROR-γt pathway, thus supporting the development of AHR and neutrophilic inflammation.

## Discussion

ILC3s have been implicated in the pathogenesis of neutrophilic asthma, but the regulatory mechanism supporting ILC3 activation in the lungs is largely unclear. In this study, we unveiled an important role for SCF/c-Kit signaling in the pathogenesis of neutrophilic asthma through the regulation of ILC3 function. We found that SCF expression was increased in the plasma, blood, and sputum samples from asthmatic patients and positively correlated to IL-17A and MPO expression, suggesting that SCF is associated with IL-17A production and neutrophilic inflammation during asthma. We identified ILC3s as potential IL-17A–producing responders to SCF in the lungs. Further experiments revealed that SCF treatment promoted ILC3 functions, including proliferation and IL-17A production, in response to IL-1β/IL-23. In contrast, the deficiency of c-Kit signaling reduced ILC3 functions and ameliorated both neutrophilic inflammation and the development of AHR in 3 distinct mouse neutrophilic asthma models. Importantly, we found that fibroblast-derived SCF is critical for optimal ILC3 activation during neutrophilic inflammation, unveiling an interplay between lung fibroblasts and ILC3s. Furthermore, pharmacological inhibition of c-Kit signaling by using imatinib was shown to ameliorate AHR and neutrophilic inflammation in our mouse models.

Previously, studies have reported elevated SCF levels in various allergic diseases, including allergic rhinitis and atopic dermatitis (68, 69). Additionally, SCF expression is increased in the lungs of murine allergic asthma models triggered by cockroach allergen and worm egg antigen (48, 70), suggesting that allergen exposure could induce SCF production. Furthermore, while SCF levels are elevated in asthmatic patients, one study found no significant difference between allergic and non-allergic asthma (40). However, in this study, we observed significantly higher SCF levels in non-Type 2 patients but not in Type 2 patients compared with healthy controls. This discrepancy may arise from differences in patient classification criteria or regional variations in sample collection, which may need further investigation. In addition, several other factors may also influence SCF expression. For instance, glucocorticoids, commonly used in asthma treatment, have been shown to decrease SCF expression in asthmatic bronchi (71). However, glucocorticoids have also been reported to potentiate IL-1β–induced SCF expression in human lung fibroblasts in the short term (72). In this study, we demonstrated that both LPS and PM_2.5_ directly upregulate SCF expression in mouse lung fibroblasts. However, whether LPS- and PM_2.5_-induced lung IL-1β production could further amplify SCF expression in both mouse and human lung fibroblasts may be an intriguing question for future investigation.

According to the literature, the SCF receptor c-Kit is expressed on multiple cell types, including mast cells, CD8^+^ T cells, and ILCs (20, 73). While SCF/c-Kit signaling has been shown to regulate mast cell and ILC2 functions, which are primarily associated with eosinophilic inflammation (42, 48), our study reveals an unexpected role for SCF in IL-17A production and neutrophilic inflammation during asthma. We observed a positive correlation between SCF and both IL-17A and MPO expression in the asthmatics, suggesting a link between SCF and neutrophilic inflammation. Notably, our further analysis revealed that, among the potential IL-17A–producing cells in the lung, ILC3s are the only cells expressing c-Kit in both humans and mice. Interestingly, although SCF alone had little effects on ILC3 functions, the combination of IL-1β/IL-23 plus SCF increased the numbers of total lung ILC3s and IL-17A–producing ILC3s compared with IL-1β/IL-23 alone. Previous studies have shown that SCF treatment enhances ILC2 cytokine production (48), but compromises CD8^+^ T Cell activation (74). Here, we demonstrated that SCF treatment upregulated the genes related to proliferation, Th17 differentiation, and the JAK-STAT signaling pathway in ILC3s. Given that STAT3 activation is important for ILC3 function (57–59), we further assessed the enrichment of the JAK-STAT3 signaling pathway in whole gene expression profiles from IL-1β/IL-23 plus SCF–treated ILC3s by using GSEA (75). Accordingly, GSEA demonstrated an upregulation of JAK-STAT signaling pathway in ILC3s treated with IL-1β/IL-23 plus SCF, compared with IL-1β/IL-23 alone. To confirm the result at the protein level in lung ILC3s, we used flow cytometry and found that SCF treatment directly enhanced AKT and STAT3 phosphorylation in lung ILC3s ex vivo. Taken together, these data demonstrated that SCF acts as a cofactor to work synergistically with IL-1β/IL-23 to promote ILC3 effector function, which is associated with the STAT3 signaling pathway. However, whether c-Kit regulates ILC3 function by itself or by the interaction with other receptors, such as IL-1R1 and IL-23R, remains to be studied. Additionally, further research into the potential crosstalk between SCF/c-Kit signaling and other pathways in ILC3s may also be necessary to better regulate ILC3 function.

We employed 2 distinct approaches, genetic c-Kit deficiency (*Kit*^W-sh^) mice and anti–c-Kit antibody treatment, to examine the involvement of c-Kit signaling in neutrophilic inflammation and ILC3 activation. Both approaches have indicated that c-Kit signaling contributes to ILC3 activation, neutrophilic inflammation, and the development of AHR. Moreover, by using BrdU incorporation, we observed a significant decrease in the frequency of BrdU-labeled lung ILC3s in *Kit*^w-sh^ mice treated with IL-1β/IL-23 compared with the WT mice, confirming the involvement of c-Kit signaling in ILC3 proliferation in vivo. However, relying solely on these approaches is insufficient, due to their potential to cause mast cell deficiency (76, 77). Therefore, we generated conditional c-Kit knockout mice by crossing *Kit*^fl/fl^ with either *Il17a*^cre/+^, *Il22*^cre/+^, or *Rorc*^cre^ mice, which are known to target ILC3s (60). By using these conditional knockout mice, these data demonstrated the cell-intrinsic role of c-Kit signaling in ILC3 activation and neutrophilic inflammation. However, given that mast cells can secrete IL-17 (78), there remains a possibility that they are also affected by our Cre models. To address this, we performed adoptive transfer of BMMCs into *Kit*^W-sh^ mice treated with IL-1β/IL-23 and found that AHR was not restored, suggesting that mast cells play a limited role in our models. Additionally, previous studies identified a c-Kit^+^ ILC2 subset exhibiting ILC3-like features, including ROR-γt expression and IL-17 production (48, 79, 80). Therefore, although our experimental models (LPS, PM2.5, and OVA/LPS) are associated with ILC3 activation and we employed multiple Cre models (*Rorc*^cre^, *Il17a*^cre^, and *Il22*^cre^) to minimize off-target effects, ILC2s may still be influenced. However, in our OVA/LPS mouse model, we did not observe a significant reduction in IL-13 levels, which is mainly secreted by ILC2s, in either *Rorc*^cre^*Kit*^fl/fl^ mice or following imatinib treatment compared with controls. This suggests that ILC2s are likely not significantly impacted by these interventions. Nevertheless, further investigation is needed to fully elucidate the potential contributions of ILC2s to the observed responses.

SCF is highly expressed in lungs for both humans and mice (81), and many cells, such as endothelial cells and fibroblasts, are reported to express SCF (73). Utilizing the published scRNA-seq dataset from mouse lungs, we identified Col1a2^+^ fibroblasts as the major cellular reservoir of SCF in the lung (63). Furthermore, an scRNA-seq dataset for asthmatic lungs from the Human Cell Atlas also revealed high SCF expression in COL1A2^+^ fibroblasts (64), suggesting fibroblasts as a significant source of SCF for ILC3 activation in both human and mouse lungs. Although a previous study has shown that fibroblasts-derived SCF promotes ILC2 activation in a mouse cockroach allergic asthma model (48), whether fibroblasts-derived SCF also plays an important role in ILC3 activation during neutrophilic inflammation remained unclear. By using the mice with fibroblast-specific SCF deletion (*Col1a2*^CreERT^*SCF*^fl/fl^), we found that the mice with fibroblast-specific SCF deletion exhibited lower neutrophilia, which is associated with diminished ILC3 activation. Furthermore, previous study has demonstrated that the expression of SCF248, but not SCF220, was increased in the lungs of allergic mice (48). Consistently, our study also revealed that both LPS and PM_2.5_ induced SCF248 expression in primary lung fibroblasts. Altogether, these findings support the idea that fibroblast-derived SCF enhances ILC3 activation during neutrophilic inflammation.

In addition, Cahill et al. showed that c-Kit inhibition by imatinib could significantly increase PC_20_ in asthmatic patients, compared with the placebo group. More interestingly, the increases in FEV_1_ were positively correlated with BAL neutrophil counts but not with eosinophil counts (45). This observation suggested a therapeutic potential of targeting SCF/c-Kit signaling in neutrophilic asthma. By using imatinib to inhibit c-Kit signaling in mouse models of neutrophilic asthma, we experimentally verified that imatinib could ameliorate AHR and ILC3 activation during neutrophilic inflammation, mirroring our observations from the genetically modified mice and the treatment with anti–c-Kit antibody. Collectively, these results underscore the therapeutic potential of targeting c-Kit signaling in neutrophilic asthma and highlight the necessity of monitoring ILC3 function and activation to gain a more comprehensive understanding of the outcomes when treating asthmatic patients with imatinib. However, in addition to targeting c-Kit, imatinib is known to inhibit other tyrosine kinases, including ABL and platelet-derived growth factor receptor (PDGFR) (82). Given that PDGFR is expressed on lung fibroblasts and lung ILC precursors (83), the potential effects of imatinib on other tyrosine kinases or cell populations may require further investigation.

Altogether, our study provides insights into the role of SCF/c-Kit signaling in promoting ILC3 activation, contributing to the pathogenesis of neutrophilic inflammation and AHR. Moreover, we demonstrated that targeting SCF/c-Kit signaling can ameliorate neutrophilic inflammation and AHR, and thus emerged as a potential therapeutic strategy for neutrophilic asthma management.

## Methods

### Sex as a biological variable.

Our study examined male and female animals, and similar findings are reported for both sexes. All animal experiments were performed with sex- and age-matched (6–8 weeks) mice. For the human study, plasma samples were collected from both male and female participants.

Specific details on the research materials and portions of the experimental protocols can be found in the Supplemental Methods.

### Human samples.

Human plasma was collected and processed at Taichung Veterans General Hospital. Briefly, blood samples from asthmatic patients or healthy controls were collected in EDTA tubes and centrifuged at 800*g* for 10 minutes to separate the plasma from the cellular components of the blood. Plasma was collected and stored in frozen aliquots. Asthmatic patients were classified based on blood eosinophil counts, with Type 2 inflammation (Type 2) defined as at least 150 eosinophils/μL and non-Type 2 inflammation (non-Type 2) as under 150 eosinophils/μL according to the Global Initiative for Asthma (GINA) criteria (84). Demographic of study subjects are detailed in Supplemental Table 1.

### Animals.

All animals were housed under specific pathogen-free conditions. C57BL/6JNarl and BALB/c mice were purchased from National Laboratory Animal Center. *Rag1^–/–^* and *Rag2^–/–^* mice were purchased from Taconic Farms. *Kit*^W-sh^, *SCF*^fl/fl^, *Kit*^fl/fl^, and *Rorc*^cre^ mice were purchased from The Jackson Laboratory. *Il17a*^cre/cre^, *Il22*^cre/cre^, and *Rag1*^–/–^*Rorc*^gfp/gfp^ mice were gifts from Dr. Jr-Wen Shui (Academia Sinica, Taipei, Taiwan).

### Mouse models of lung neutrophilic inflammation.

For cytokine stimulation, mice intranasally (i.n.) received 0.1 μg of IL-1β/IL-23 and/or SCF (Biolegend) for three consecutive days (Days 0–2). On day 6, AHR and airway inflammation were assessed and lung samples were collected.

For LPS treatment, mice i.n. received 2 μg of LPS (L4268; Sigma-Aldrich) for four consecutive days (Days 0–3). AHR and airway inflammation were assessed and lung samples were collected on day 4.

For PM_2.5_ treatment, mice were i.n. treated with 200 μg of PM_2.5_ (SRM2786; Sigma-Aldrich) for three consecutive days (Days 0–2). AHR and airway inflammation were assessed and lung samples were collected on day 6.

For OVA/LPS treatment, the model was established following previously described protocols (35, 67, 85). Briefly, mice were i.n. sensitized with 75 μg of OVA (Worthington Biochemical Co.) and 10 μg of LPS on days 0, 1, 2, and 7, and then i.n. challenged with 100 μg of OVA on days 14, 15, 21, and 22. AHR and airway inflammation were assessed and lung samples were collected on day 23.

For imatinib treatment, mice intraperitoneally (i.p.) received imatinib (100 mg/kg) to block c-Kit signaling on either day 1 (For IL-1β/IL-23 and PM_2.5_ treatment), days 1 and 3 (For LPS treatment), or days 14 and 21 (For OVA/LPS treatment). AHR and airway inflammation were assessed and lung samples were collected on the indicated day.

### Lung sample preparation and cell isolation.

Lung tissues were minced and incubated in DMEM containing 0.1% (vol/vol) DNase I (Worthington Biochemicals) and 1.6 mg/mL collagenase IV (Worthington Biochemicals) for 30 minutes at 37°C. Tissue aggregates were then dissociated with an 18-gauge needle and lung tissues were further incubated at 37°C for 15 minutes. Tissues were filtered through a 70 μm mesh to obtain single-cell suspensions. ACK lysing buffer (Gibco) was used for red blood cell lysis. The single-cell suspensions were then used for flow cytometry.

### Flow cytometry.

Single-cell suspensions were stained with fixable viability dye (65-0865-14, eBioscience), followed by incubation with anti-mouse CD16/32 blocking antibody (93, Biolegend). Cells were then stained with the appropriate surface antibodies at 4°C for 30 minutes. For intracellular cytokine staining, cells were stimulated with 100 ng/mL phorbol 12-myristate 13-acetate, 1 μg/mL ionomycin in RPMI 1640 supplemented with 10% FBS for 4 hours, and GolgiStop (BD Biosciences) was added in the last 1 hour of incubation. Then, cells were fixed and permeabilized with Foxp3/Transcription Factor Staining Buffer Set (eBioscience) according to the manufacturer’s instructions. Cells were then incubated with intracellular antibodies at 4°C for 30 minutes.

For BrdU detection, cells from lung samples were stained for fixable viability dye (65-0865-14, eBioscience), followed by incubation with anti-mouse CD16/32 blocking antibody (93, Biolegend). Next, cells were incubated with the appropriate surface antibodies at 4°C for 30 minutes, fixed, and permeabilized with Foxp3/Transcription Factor Staining Buffer Set (eBioscience) according to the manufacturer’s instructions, and then treated with 30 μg of DNase I (Sigma-Aldrich) and incubated at 37°C for 1 hour for exposure of BrdU-labeled epitopes. Then, cells were stained with anti-ROR-γt (AFKJS-9; eBioscience) and anti-BrdU (3D4; Biolegend) at 4°C for 30 minutes.

For analysis of intracellular signaling in lung ILC3s, sorted lung ILCs will be serum-starved for 2 hours prior to stimulation with IL-1β/IL-23 (10 ng/mL; Biolegend) in the presence or absence of SCF (100 ng/mL; Biolegend) for 10 minutes at 37°C. Cells were then be fixed with 4% paraformaldehyde (PFA) at 4°C for 30 minutes before permeabilization in 90% methanol at 4°C for 30 minutes. Then, cells were stained with anti-ROR-γt (AFKJS-9; eBioscience), anti-pAKT (pS473) (D9E; Cell Signaling Technology) and anti-pSTAT3 (pY705) (D3A7; Cell Signaling Technology) at room temperature for 30 minutes.

LSR II (BD Biosciences) was used for cell analysis and FlowJo software (Version 10.1; TreeStar) was used for data analysis.

### Lung ILC sorting.

Lung ILCs were sorted from *Rag2*^–/–^ mice. Briefly, total lung cells were resuspended in 33% Percoll (GE Healthcare) and centrifuged at 800*g* for 30 minutes to obtain mononuclear cells prior to surface staining with the appropriate antibodies. ILCs were sorted as CD45^+^Lin^-^Thy1.2^+^ cells (Supplemental Figure 1B). Lineage markers used are CD3e, CD19, FcεRI, F4/80, CD11b, CD11c, and CD49b. Sorting was performed with a FACSAria IIIu cell sorter (BD Biosciences) with a sorting purity of greater than 95%. Cells were maintained in RPMI 1640 supplemented with 10% FBS, IL-2, and IL-7 (all at 10 ng/mL).

### Small intestine ILC3 sorting.

Single-cell suspensions of intestinal lamina propria were prepared as previously described (86, 87). Small intestine ILC3s were isolated from *Rag1*^–/–^ mice. Briefly, fats were removed from small intestines. Then, small intestines were opened longitudinally and washed with cold PBS. Intestines were then incubated at 37°C for 40 minutes in PBS containing 5 mM EDTA to remove intestinal epithelium. Tissues were washed and then digested with 0.8 mg/mL collagenase IV (Worthington Biochemicals) in RPMI 1640 for 40 minutes. Cell suspensions were filtered through 70 μm mesh and purified through density gradient centrifugation using Percoll (GE Healthcare). Then, ILC3s were sorted as CD45^int^Thy1.2^hi^Lin^-^KLRG1^-^ cells (Supplemental Figure 1C). Lineage markers used were CD3e, CD19, FcεRI, F4/80, CD11b, CD11c, and CD49b. Sorting was performed with a FACSAria IIIu cell sorter (BD Biosciences). Cells were maintained in RPMI 1640 supplemented with 10% FBS, IL-2, and IL-7 (all at 10 ng/mL).

### Statistics.

Statistical analyses were performed using Prism software (v9.5.1). For the comparisons between 2 groups, 2-tailed Student’s *t* test for unpaired or paired data was used. For multiple groups comparisons, 1-way ANOVA and 2-way ANOVA were used. A *P* value of less than 0.05 was considered significant. All in vivo experiments were repeated at least twice and combined data are presented as mean ± SEM. All ex vivo experiments were repeated independently 3 times and data are presented as mean ± SEM. Statistical details can be found in the figure legends.

### Study approval.

The human research protocol was approved by Academia Sinica Institutional Review Board for Biomedical Science Research (AS-IRB-BM-22063 and AS-IRB-BM-23029). Informed consent was obtained from participants before sample collection. All mouse experiment protocols were approved by Academia Sinica Institutional Animal Care and Use Committee (IACUC, 20-11-1549), and all experiments were performed according to the guidelines of IACUC.

### Data availability.

All data associated with this study are present in the paper or the Supplemental material. RNA-seq data generated in this study have been deposited in the Gene Expression Omnibus database under accession number GSE275251. All data values are reported in the Supporting Data Values file. This study analyzes publicly available microarray data for asthmatic blood (GSE69683) and induced sputum (GSE76262) samples. scRNA-seq data for mouse lung cells are available on GEO (GSE109774). scRNA-seq data for Human lung T cells and ILCs and asthmatic lung cells are available on CELLxGENE (https://cellxgene.cziscience.com/collections/62ef75e4-cbea-454e-a0ce-998ec40223d3) and the Human Cell Atlas (https://asthma.cellgeni.sanger.ac.uk), respectively.

## Author contributions

JSS and YJC conceptualized the study and designed experiments. JSS, ACYL, WCH, KCW, and PYC performed the experiments and conducted analyses. Human sample acquisition was performed by WCH. Bioinformatics analysis was performed by YMC. JSS and ACYL wrote the original draft of the manuscript, which was reviewed and edited by YJC. YJC acquired funding and supervised the study.

## Supplementary Material

Supplemental data

Supporting data values

## Version 1 07/17/2025

In-Press Preview

## Version 2 08/15/2025

Electronic publication
