# Supplementary material for: Pulmonary fibroblast-derived stem cell factor promotes neutrophilic asthma by augmenting IL-17A production from ILC3s
Source: J Clin Invest. 2025 Jul 17;135(16):e187372. doi: 10.1172/JCI187372 (PMC12352899; doi:10.1172/JCI187372)
Supplement: Supplemental data [file jci-135-187372-s172.pdf]

Supplemental material for

Pulmonary fibroblast-derived stem cell factor promotes neutrophilic asthma by augmenting IL-17A production from ILC3s

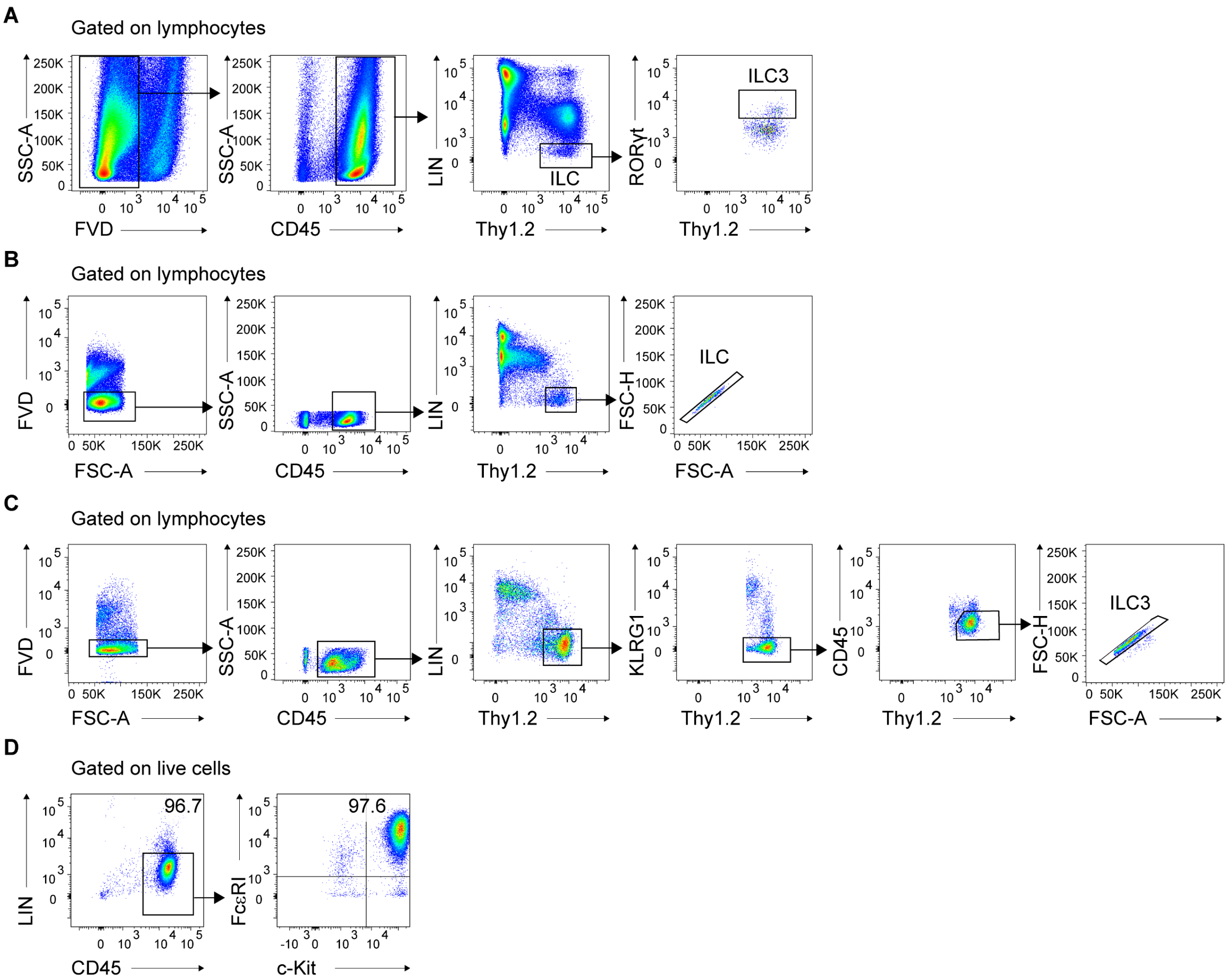

Supplemental Figure 1. Gating strategies.

(**A**) Gating strategy for ILC3 in lung tissues. (**B**) Gating strategy for ILC in lung tissues. (**C**) Gating strategy for ILC3 in small intestine tissues. (**D**) Flow cytometric analysis of bone-marrow derived mast cell after 5 weeks of culture. FVD, fixable viability dye; ILC, innate lymphoid cell; ILC3, group3 innate lymphoid cell.

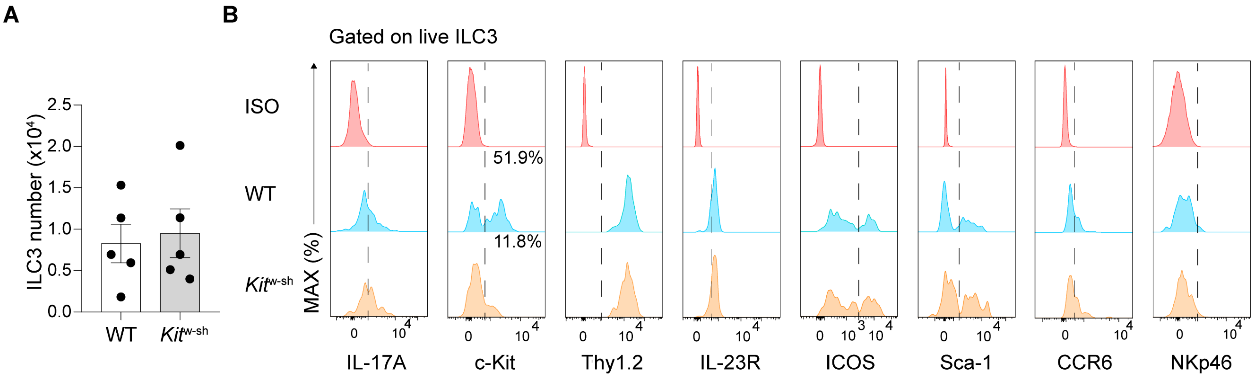

Supplemental Figure 2. Phenotypical analysis of ILC3 from WT and *Kit*$^{\text{W-sh}}$ mice.

(**A-B**) C57BL/6 (WT) and c-Kit deficient (*Kit*$^{\text{W-sh}}$) mice were used for following analysis. (**A**) Numbers of lung ILC3s (CD45$^+$Thy1.2$^+$Lin$^-$RORγt$^+$) (**B**) Representative histogram of indicated markers in ILC3s (CD45$^+$Thy1.2$^+$Lin$^-$RORγt$^+$). n = 5 per group. Data are means ± SEM and are representative of at least 2 independent experiments. Significance was determined by two-tailed unpaired Student's t test (**A**).

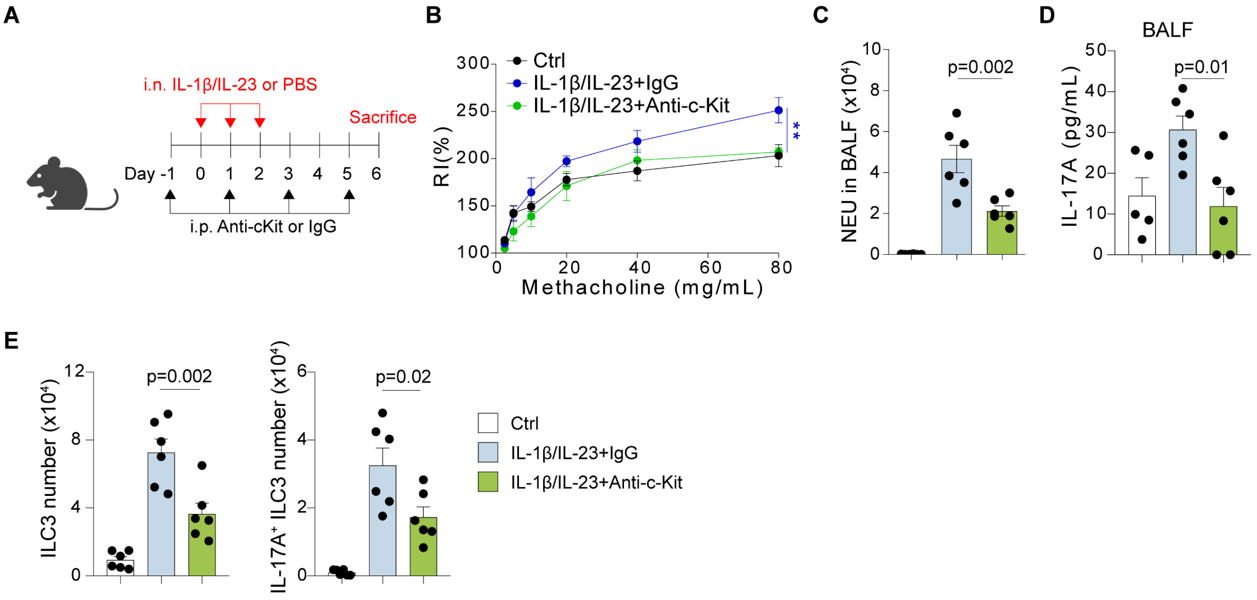

Supplemental Figure 3. Blocking c-Kit signaling ameliorates IL-1β/IL-23-induced neutrophilic inflammation, AHR and ILC3 responses.

(**A-E**) C57BL/6 (WT) mice intraperitoneally (i.p.) injected either control IgG or anti-c-Kit antibody every other day and intranasally (i.n.) received IL-1β/IL-23 for 3 consecutive days (Day 0-2). Mice were sacrificed on day 6 for following analysis. (**A**) Experimental scheme. (**B**) Lung resistance in response to increasing doses of methacholine. (**C**) Numbers of neutrophils (NEU) in BALF. (**D**) IL-17A protein levels in BALF. (**E**) Numbers of lung ILC3s ($CD45^+Thy1.2^+Lin^-ROR\gamma t^+$) and IL-17A$^+$ ILC3s ($CD45^+Thy1.2^+Lin^-ROR\gamma t^+IL\text{-}17A^+$). n = 5-6 per group. Data are means ± SEM and are representative of at least 2 independent experiments. Significance was determined by two-way ANOVA (**B**) and one-way ANOVA (**C-E**); * P < .05; ** P < .01; *** P < .001.

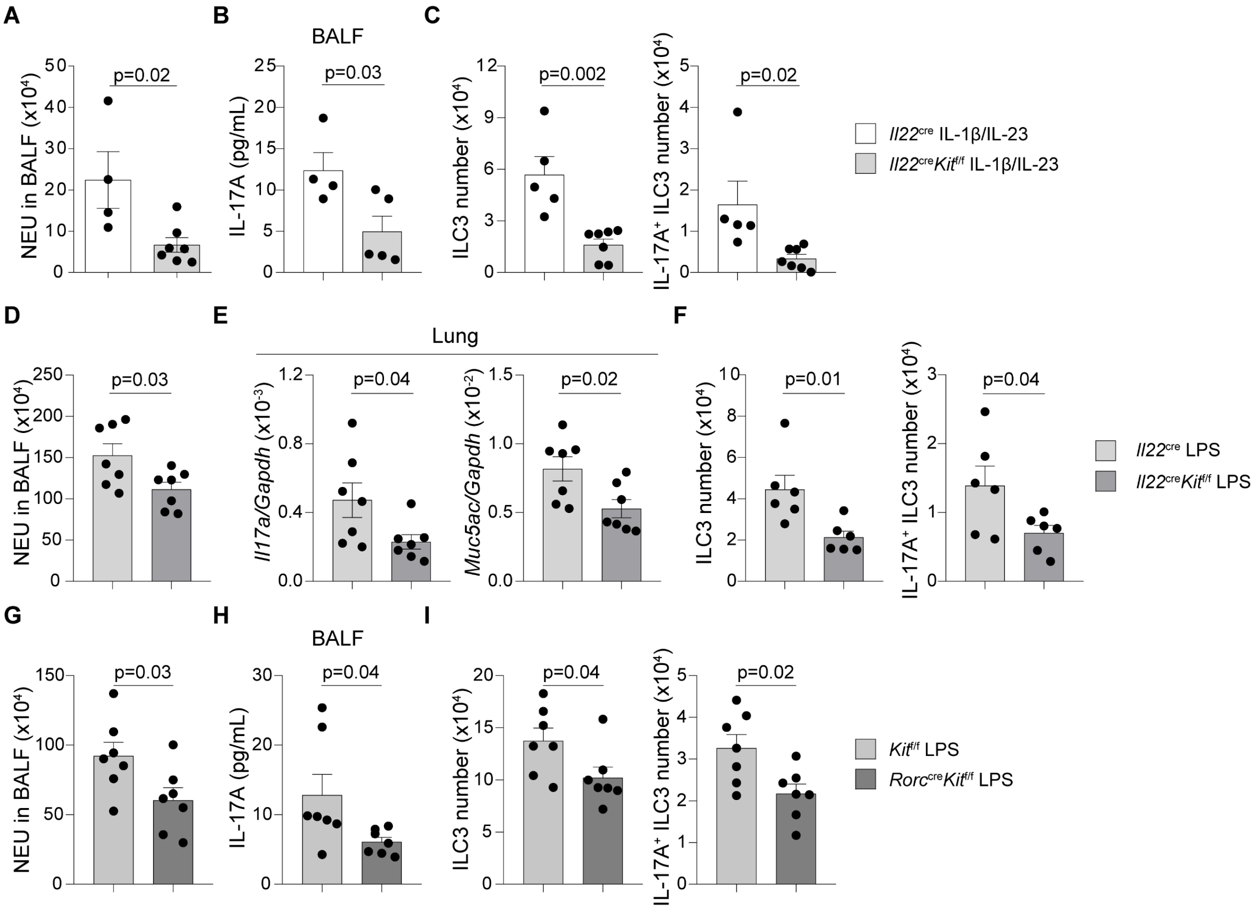

Supplemental Figure 4. Conditional deletion of c-Kit in ILC3s alleviates neutrophilic inflammation and ILC3 responses.

(**A-C**) *Il22*$^{cre/+}$ and *Il22*$^{cre/+}$*Kit*$^{f/f}$ mice i.n. received IL-1β and IL-23 for 3 days (Day 0-2) and were sacrificed on day 6 for following analysis. (**A**) Numbers of NEU in BALF. (**B**) IL-17A protein levels in BALF. (**C**) Numbers of lung ILC3s (CD45$^+$Thy1.2$^+$Lin$^-$RORγt$^+$) and IL-17A$^+$ ILC3s (CD45$^+$Thy1.2$^+$Lin$^-$RORγt$^+$IL-17A$^+$). n = 4-7 per group. (**D-F**) *Il22*$^{cre/+}$ and *Il22*$^{cre/+}$*Kit*$^{f/f}$ mice i.n. received LPS for 4 days (Day 0-3) and were sacrificed on day 4 for following analysis. (**D**) Numbers of NEU in BALF. (**E**) mRNA levels of *Il17a* and *Muc5ac* in lung lysates. (F) Numbers of lung ILC3s (CD45$^+$Thy1.2$^+$Lin$^-$RORγt$^+$) and IL-17A$^+$ ILC3s (CD45$^+$Thy1.2$^+$Lin$^-$RORγt$^+$IL-17A$^+$). n = 6-7 per group. (**G-I**) *Kit*$^{f/f}$ and *Rorc*^{cre}*Kit*$^{f/f}$ mice i.n. received LPS for 4 days (Day 0-3)

and were sacrificed on day 4 for following analysis. (**G**) Numbers of NEU in BALF. (**H**) IL-17A protein levels in BALF. (**I**) Numbers of lung ILC3s ($CD45^+Thy1.2^+Lin^-ROR\gamma t^+$) and IL-17A$^+$ ILC3s ($CD45^+Thy1.2^+Lin^-ROR\gamma t^+IL\text{-}17A^+$). n = 7 per group. Data are means ± SEM and are representative of at least 2 independent experiments. Significance was determined by two-tailed unpaired Student's t test.

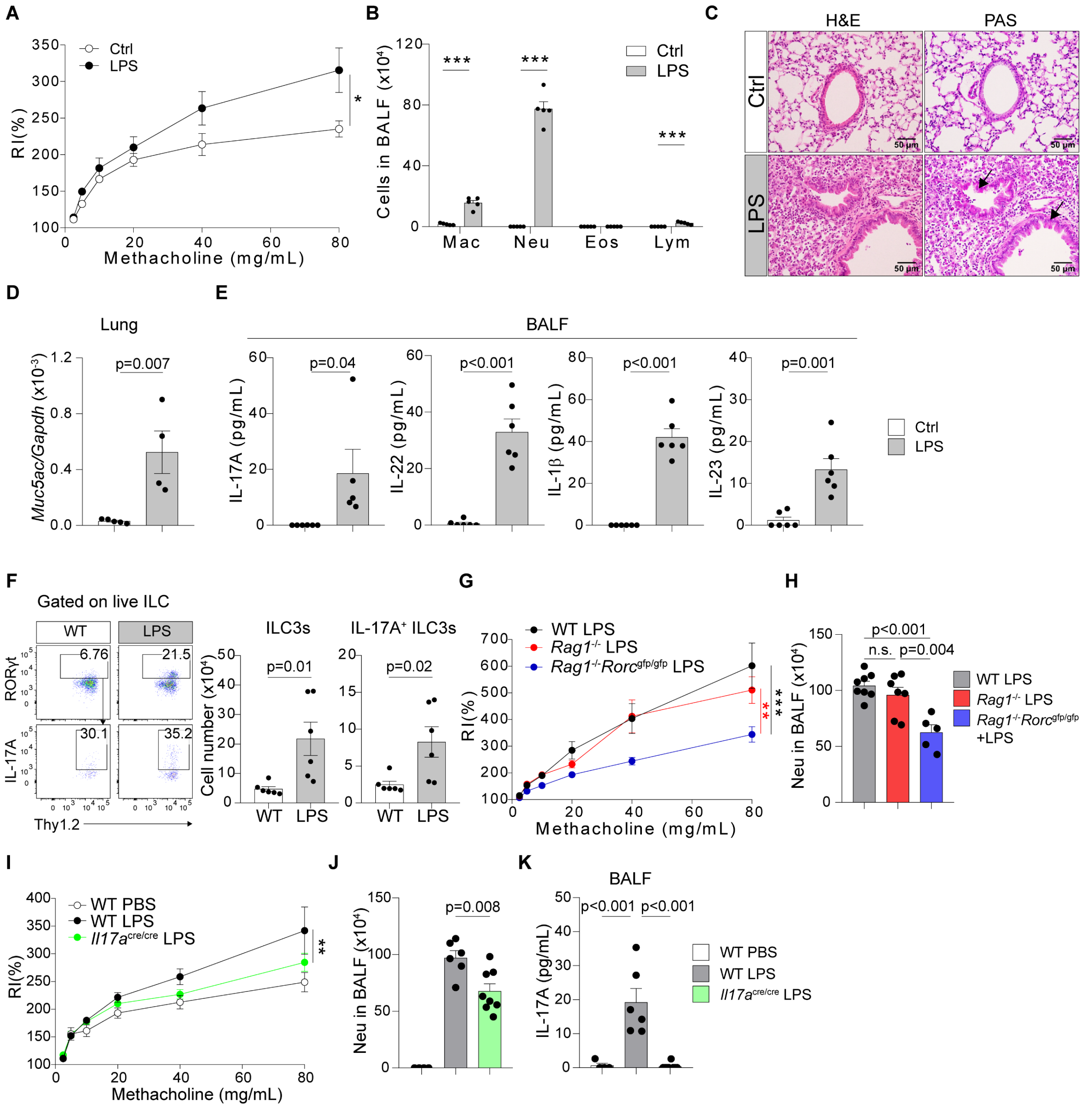

Supplemental Figure 5. ILC3 is required for optimal LPS-induced neutrophilic airway inflammation and AHR.

(**A-F**) C57BL/6 (WT) mice intranasally (i.n.) received LPS for 4 days (Day 0-3) and were sacrificed on day 4 for following analysis. (**A**) Lung resistance in response to increasing doses of methacholine. (**B**) Cellular composition in BALF. Mac, macrophage; Neu, neutrophil; Eos,

eosinophil; Lym, lymphocyte. (**C**) Representative H&E staining and PAS staining of the lung from control and LPS-treated mice. The arrowheads indicated the mucus deposition. Scale bars, 50μm. (**D**) *Muc5ac* mRNA levels in lung lysates. (**E**) IL-17A, IL-22, IL-1β and IL-23 protein levels in BALF. (**F**) Flow cytometry analysis of ILC3s and numbers of lung ILC3s ($CD45^{+}Thy1.2^{+}Lin^{-}ROR\gamma t^{+}$) and IL-$17A^{+}$ ILC3s ($CD45^{+}Thy1.2^{+}Lin^{-}ROR\gamma t^{+}IL\text{-}17A^{+}$). n = 4-6 per group. (**G-H**) WT, $Rag1^{-/-}$ and $Rag1^{-/-}Rorc^{gfp/gfp}$ mice i.n. received LPS for 4 days (Day 0-3) and were sacrificed on day 4 for following analysis. (**G**) Lung resistance in response to increasing doses of methacholine. (**H**) Numbers of neutrophils (NEU) in BALF. n = 5-8 per group. (**I-K**) WT and $Il17a^{cre/cre}$ mice i.n. received LPS for 4 days (Day 0-3) and were sacrificed on day 4 for following analysis. (**I**) Lung resistance in response to increasing doses of methacholine. (**J**) Numbers of NEU in BALF. (**K**) IL-17A protein levels in BALF. n = 4-8 per group. Data are means ± SEM and are representative of at least 2 independent experiments. Significance was determined by two-way ANOVA (**A**, **G**, and **I**), multiple t test (**B**), two-tailed unpaired Student's t test (**D-F**) and one-way ANOVA (**H**, **J**, and **K**); * $P < .05$; ** $P < .01$; *** $P < .001$.

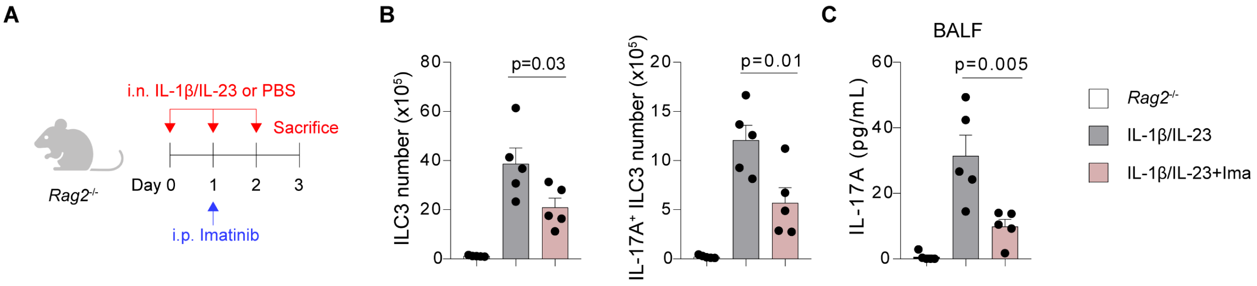

Supplemental Figure 6. Imatinib reduces ILC3 responses independently of adaptive immunity.

(**A-C**) *Rag2*$^{-/-}$ mice intranasally (i.n.) received IL-1β/IL-23 for 3 consecutive days (Day 0-2) and intraperitoneally (i.p.) received imatinib (Ima) on day 1. Mice were sacrificed on day 3 for following analysis. (**A**) Experimental scheme. (**B**) Numbers of lung ILC3s (CD45$^+$Thy1.2$^+$Lin$^-$ RORγt$^+$) and IL-17A$^+$ ILC3s (CD45$^+$Thy1.2$^+$Lin$^-$RORγt$^+$IL-17A$^+$). (**C**) IL-17A protein levels in BALF. n = 5 per group. Data are means ± SEM and are representative of at least 2 independent experiments. Significance was determined by one-way ANOVA (**B-C**).

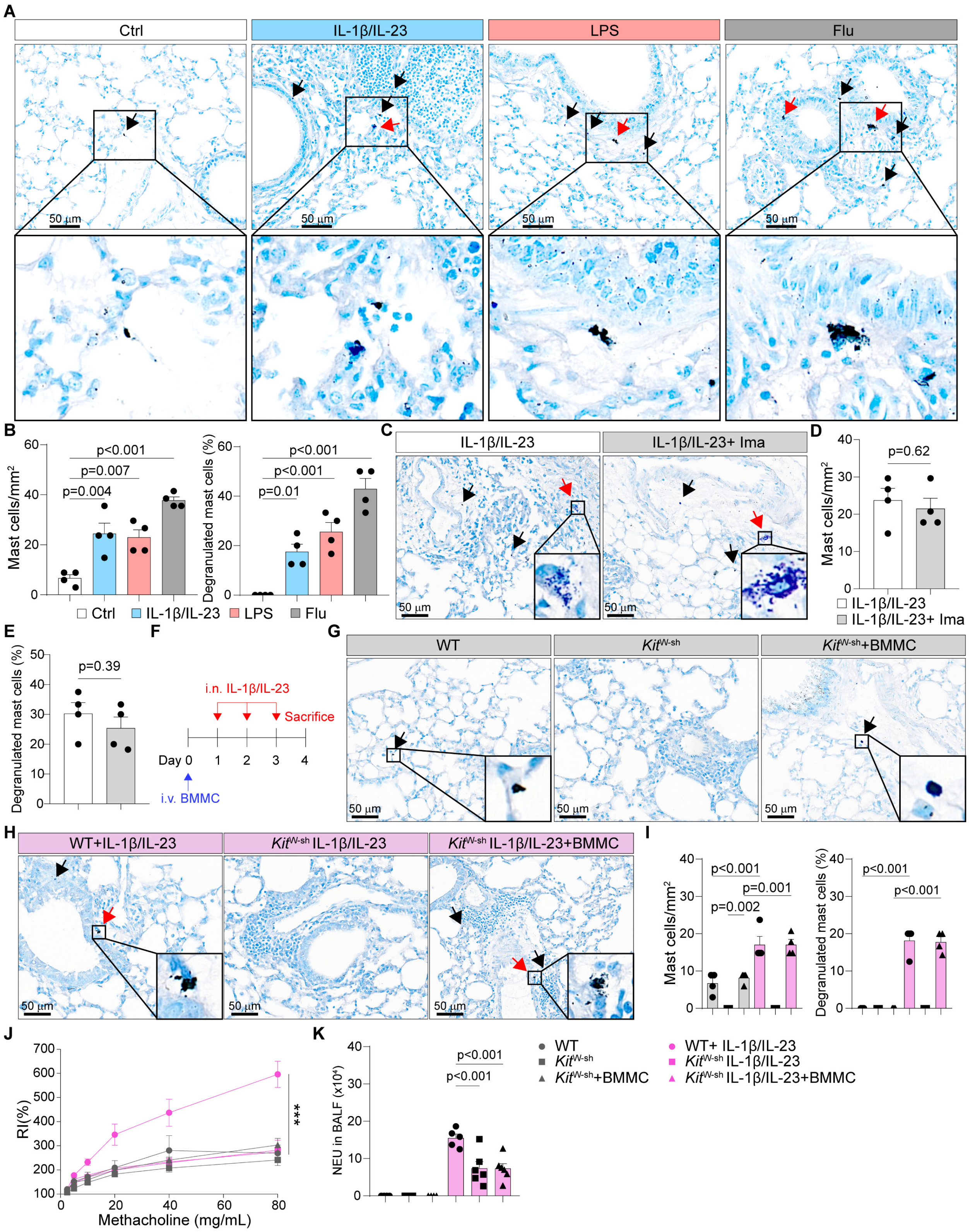
A
Ctrl
IL-1β/IL-23
LPS
Flu
50 μm
B
Mast cells/mm²
p<0.001
p=0.007
p=0.004
Degranulated mast cells (%)
p<0.001
p<0.001
p=0.01
Ctrl
IL-1β/IL-23
LPS
Flu
C
IL-1β/IL-23
IL-1β/IL-23+ Ima
D
Mast cells/mm²
p=0.62
IL-1β/IL-23
IL-1β/IL-23+ Ima
E
Degranulated mast cells (%)
p=0.39
F
i.n. IL-1β/IL-23
Sacrifice
Day 0
1
2
3
4
i.v. BMMC
G
WT
Kit^W-sh
Kit^W-sh+BMMC
H
WT+IL-1β/IL-23
Kit^W-sh IL-1β/IL-23
Kit^W-sh IL-1β/IL-23+BMMC
I
Mast cells/mm²
p<0.001
p=0.001
p=0.002
Degranulated mast cells (%)
p<0.001
p<0.001
J
RI(%)
***
Methacholine (mg/mL)
K
NEU in BALF (x10⁴)
p<0.001
p<0.001
WT
Kit^W-sh
Kit^W-sh+BMMC
WT+ IL-1β/IL-23
Kit^W-sh IL-1β/IL-23
Kit^W-sh IL-1β/IL-23+BMMC

Supplemental Figure 7. Reconstitution of mast cells fails to restore AHR in IL-1β/IL-23-treated *Kit*$^{W\text{-}sh}$ mice.

(**A-B**) C57BL/6 (WT) mice intranasally (i.n.) received influenza, IL-1β/IL-23 or LPS. (**A**) Representative images of toluidine blue-stained lung sections. (**B**) Numbers of mast cells and percentage of degranulated mast cells in the lungs. n = 4 per group. (**C-E**) C57BL/6 (WT) mice i.n. received IL-1β/IL-23 for 3 consecutive days (Day 0-2) and intraperitoneally (i.p.) received imatinib (Ima) on day 1. Mice were sacrificed on day 3 for following analysis. (**C**) Representative images of toluidine blue-stained lung sections. (**D**) Numbers of mast cells in the lungs. (**E**) Percentage of degranulated mast cells in the lungs. n = 4 per group. (**F-K**) C57BL/6 (WT) mice were intravenously injected with 10^7 BMMCs on day 0 and i.n. received IL-1β/IL-23 for 3 consecutive days (Day 1-3). (**F**) Experimental scheme. (**G-H**) Representative images of toluidine blue-stained lung sections. (**I**) Numbers of mast cells and percentage of degranulated mast cells in the lungs. (**J**) Lung resistance in response to increasing doses of methacholine. (**K**) Numbers of neutrophils (NEU) in BALF. n = 4-6 per group. Data are means ± SEM and are representative of at least 2 independent experiments. For **A**, **C** and **G-H**, black arrows indicated mast cells and red arrows indicated degranulated mast cells. Scale bars, 50μm. Significance was determined by one-way ANOVA (**B, I and K**), two-tailed unpaired Student's t test (**D-E**) and two-way ANOVA (**J**).

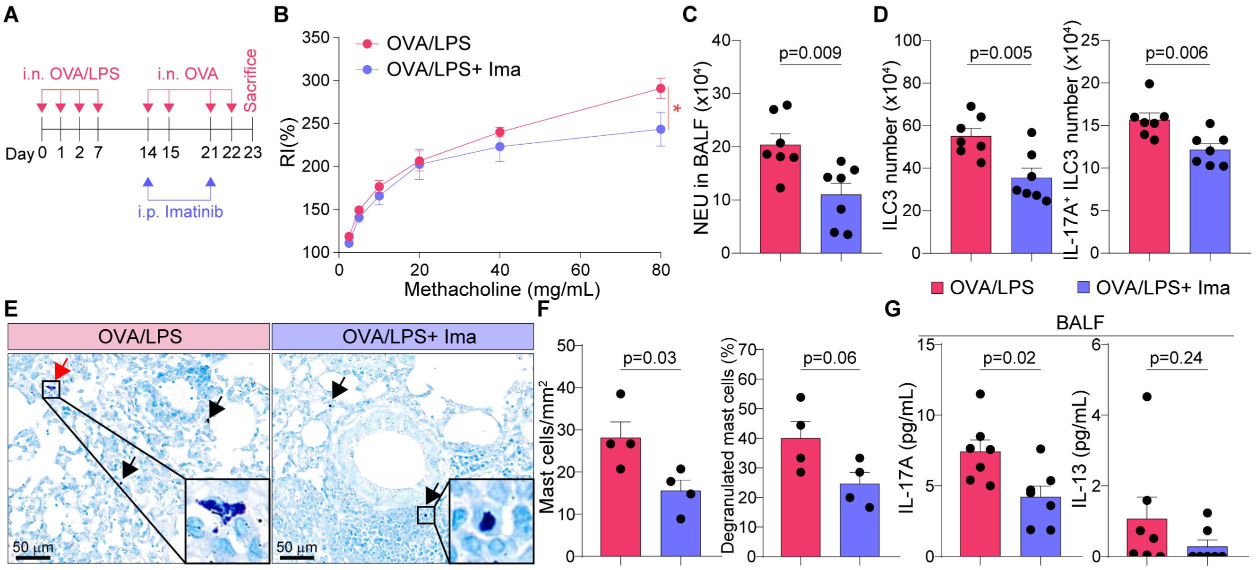

Supplemental Figure 8. Imatinib reduces OVA/LPS-induced AHR and ILC3 responses.

(**A-G**) C57BL/6 (WT) mice were i.n. administered OVA/LPS on day 0, 1, 2, and 7, followed by OVA on day 14, 15, 21, and 22, and i.p. received imatinib on day 14 and 21. Mice were sacrificed on day 23 for following analysis. (**A**) Experimental scheme. (**B**) Lung resistance in response to increasing doses of methacholine. (**C**) Numbers of neutrophils (NEU) in BALF. (**D**) Numbers of lung ILC3s and IL-17A$^+$ ILC3s. (**E**) Representative images of toluidine blue-stained lung sections. Black arrows indicated mast cells and red arrows indicated degranulated mast cells. Scale bars, 50µm. (**F**) Numbers of mast cells and percentage of degranulated mast cells in the lungs. (**G**) IL-17A and IL-13 protein levels in BALF. n = 4-7 per group. Data are means ± SEM and are representative of at least 2 independent experiments. Significance was determined by two-way ANOVA (**B**) and two-tailed unpaired Student's t test (**C-D** and **F-G**); * P < .05; ** P < .01; *** P < .001.

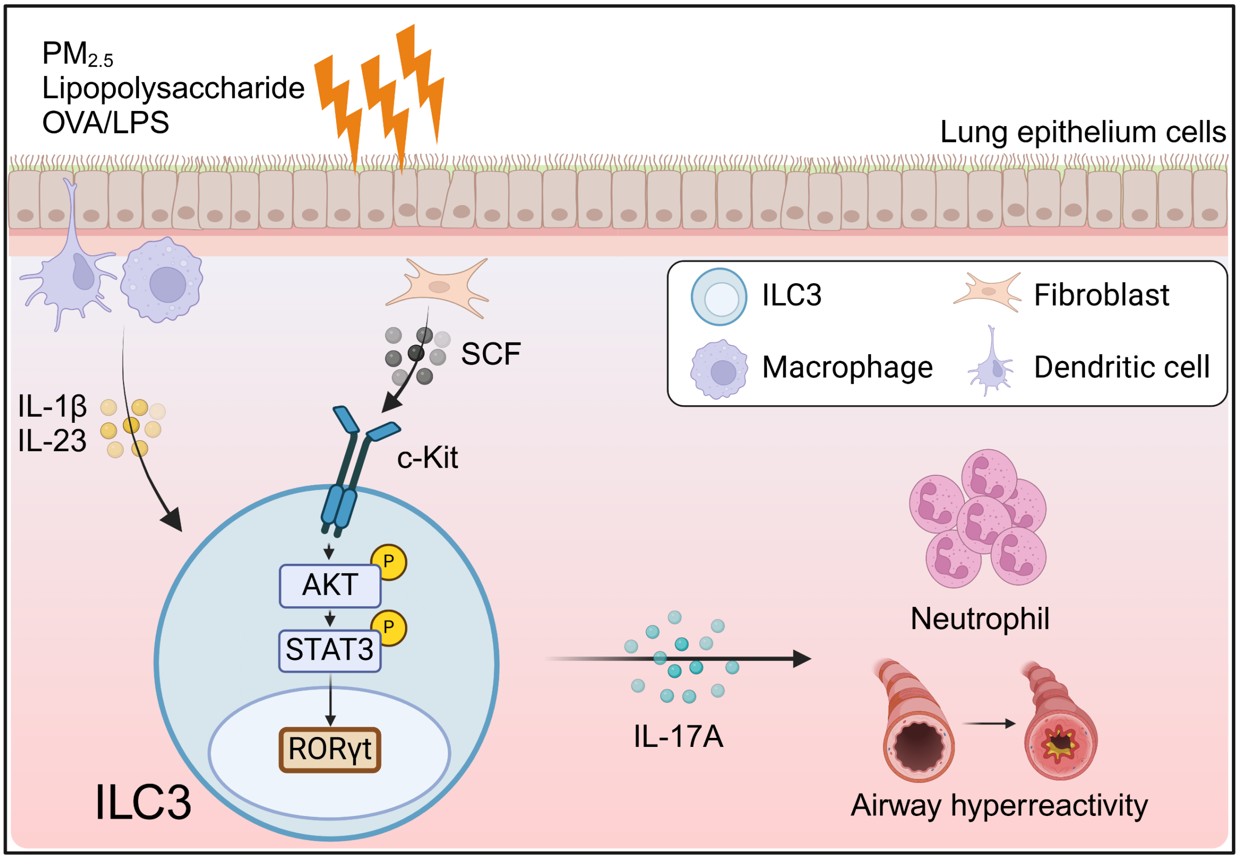

Graphical abstract. Proposed model of SCF/c-Kit signaling in ILC3 function and neutrophilic asthma.

During $PM_{2.5}$, LPS or OVA/LPS-induced inflammation, lung fibroblasts produce SCF for optimal ILC3 activation, which is associated with enhanced AKT/STAT3/RORγt pathway. Consequently, activated ILC3s secrete IL-17A and contribute to the development of AHR and neutrophilic inflammation.

122 **Supplemental Table 1. Demographic of study subjects.**

Characteristic	Healthy	Asthma	
		Type 2	non-Type 2
No. of subjects	11	10	7
Male/Female	4/7	3/7	4/3
Age (years)	38.73 ± 8.51	58.9 ± 13.37	45.29 ± 18.34
BMI (kg/m^2)	NA	28.44 ± 5.91	24.84 ± 3.04
Allergic rhinitis, n (%)	NA	4 (40%)	5 (71%)
Atopic dermatitis, n (%)	NA	1 (10%)	0
**Race**			
Asian	11 (100%)	10 (100%)	7 (100%)
**Spirometry**			
FEV1 (L)	NA	1.53 ± 0.86	2.21 ± 1.14
FEV1 (%)	NA	53.18 ± 17.81	76.09 ± 28.09
FVC (L)	NA	2.56 ± 1.06	3.28 ± 0.99
FVC (%)	NA	73.83 ± 12.69	94.39 ± 15.74
FEV1/FVC ratio	NA	58.20 ± 13.17	68.71 ± 16.88
**Smoking history**			
Never, n (%)	11 (100%)	8 (80%)	6 (86%)
Ex, n (%)	0	2 (20%)	1 (14%)
Current, n (%)	0	0	0
Blood eosinophil count (per μL)	NA	281.91 ± 86.1	66.68 ± 54.81

Data are presented as means ± standard deviation (SD) or as no. (%). BMI, body mass index; FEV1, forced expiratory volume in the first second; FVC, forced vital capacity; NA, not applicable.

123

124

Supplemental Table 2. Primer sequences used in this study

	Forward primer (5'-3')	Reverse primer (5'-3')
m*Gapdh*	AGGTCGGTGTGAACGGATTTG	TGTAGACCATGTAGTTGAGGTCA
m*Il17a*	TCCAGAAGGCCCTCAGACTA	ACACCCACCAGCATCTTCTC
m*Muc5ac*	CCATGCAGAGTCCTCAGAACAA	TTACTGGAAAGGCCCAAGC
m*SCF248*	GTTGCAGCCAGCTCCCTTA	CCATTGTAGGCCCGAGTCTT
m*SCF220*	TCCCGAGAAAGGGAAAGCC	CGAAATGAGAGCCGGCAATG

Supplemental Methods

Antibodies and reagents

For flow cytometry, the following mouse antibodies were used: CD45 (30-F11; Biolegend), Thy1.2 (53-2.1; Biolegend), KLRG1 (2F1; Biolegend), BrdU (3D4; Biolegend), c-Kit (2B8; Biolegend), ICOS (c398.4A; Biolegend), NKp46 (29A1.4; Biolegend), CCR6 (29-2L17; Biolegend), IL-17A (eBio17B7; eBioscience), IL-23R (12B2B64; Biolegend), RORγt (AFKJS-9; eBioscience), Sca-1 (D7; Biolegend), pAKT (D9E; Cell Signaling Technology), pSTAT3 (D3A7; Cell Signaling Technology), lineage (Lin) cocktail for ILCs including CD3e (145-2C11; Biolegend), CD11b (M1/70; Biolegend), CD11c (N418; Biolegend), CD19 (6D5; Biolegend), CD49b (DX5; Biolegend), F4/80 (BM8; Biolegend), FcεRI (MAR-1; Biolegend), and lineage (Lin) cocktail for mast cell including Thy1.2 (53-2.1; Biolegend), CD3e (145-2C11; Biolegend), CD11b (M1/70; Biolegend), CD11c (N418; Biolegend), CD19 (6D5; Biolegend), F4/80 (BM8; Biolegend).

Influenza virus infection

Mice were infected as previously described (1). Briefly, mice were intranasally infected with 12,000 plaque-forming units (pfu) of influenza A virus (strain Mem/71 (H3N1)) on day 0. Lung sample were collected on day 7 for following analysis.

Anti-c-Kit antibody treatment

Mice were i.n. treated with 0.1ug of IL-1β and IL-23 for three consecutive days (Day 0 to 2). Anti–c-Kit (250ug; BE0293, BioXcell) or control IgG antibodies (250ug; BE0090, BioXcell) were i.p. injected into mice starting on day -1 and continuing every other day until the end of the experiment (Day 6).

In vivo BrdU incorporation

Mice were i.n. treated with 0.1ug of IL-1β and IL-23 (Biolegend) for three consecutive days (Day 0 to 2), and then with 0.8mg of BrdU (Biolegend) from day 3 to 5. On day 6, mice were sacrificed and lung samples were processed for ILC3 proliferation analysis.

AHR measurement

Mice were anesthetized with pentobarbital (Sigma-Aldrich) at 100 mg/kg body weight. Mice were then tracheotomized and mechanically ventilated at a tidal volume of 0.20 mL and a frequency of 150 breaths/min using FinePointe RC System (Buxco Research Systems). Lung function was assessed by direct measurement of lung resistance (R_L) in response to increasing methacholine (Sigma-Aldrich) concentrations (2.5-80 mg/mL).

BALF collection

BALF was collected by putting a catheter into trachea and instilling 500 μL of PBS for first lavage. Second lavage is collected by instilling 1 mL 2% FCS/PBS. BALF cells was pelleted by centrifugation at 400g for 5 minutes and pooled. BALF cells were resuspended in 2% FCS PBS

and fixed onto cytospin slides through centrifugation. Slides were stained with Diff-Quik solution (Polysciences), and BALF differential cell counts were performed. Supernatants of first lavage were used to determine cytokine production in BALF.

Lung histology

Lungs were fixed with 4% paraformaldehyde, and dehydrated sequentially with 30%, 50%, and 70% ethanol, followed by paraffin embedding. Paraffin-embedded lung sections were stained with hematoxylin and eosin (H&E) or periodic acid-Schiff (PAS). For toluidine blue staining, sections were stained using toluidine blue solution (Servicebio) for 2 mins and then sections were rinsed in distilled water, dehydrated through a graded ethanol series, cleared in xylene, and mounted using a mounting medium (Sigma-Aldrich). For mast cell quantification, at least three randomly selected fields per mouse were counted. Images were acquired with an Olympus CX31 microscope (Olympus Corp.).

ELISA

The concentrations of mouse IL-1β, IL-23 and IL-22 in BALF and human SCF in plasma were measured by using ELISA MAX Deluxe set from BioLegend according to manufacturer's instructions. Mouse IL-17A and IL-13 in BALF and human IgE were quantified by ELISA kit purchased from Invitrogen.

Lung fibroblast isolation and stimulation

Lung fibroblasts were isolated as previously described (2). Briefly, lung tissues were minced and incubated in DMEM containing 0.1% (vol/vol) DNase I (Worthington Biochemicals) and 1 mg/ml

collagenase A (Roche) for 30 minutes at 37 °C. Tissues were dissociated with an 18-gauge needle and lung tissues were then incubated at 37°C for 15 minutes. Tissues were filtered through a 70-mm mesh to obtain single-cell suspensions, and then cultured in DMEM supplemented with 15% FBS. Cells were used for experiments after one passage. For stimulation, fibroblasts were treated with either LPS or $PM_{2.5}$ (SRM2786; Sigma-Aldrich) for 24 hours. RNA samples were collected by using TRIzol reagent.

RNA isolation and quantitative real-time PCR (qRT-PCR)

Total RNA from lung tissue was extracted by using Direct-zol RNA Miniprep (Zymo Research). 2ug of total RNA was used to synthesize cDNA by using the High-Capacity cDNA Reverse Transcription Kit (Applied Biosystems), and quantitative real-time PCR was performed on TOptical 96 Real-time PCR Thermal Cycler (Biometra). Gene-specific PCR products were amplified by using qPCR SYBR Green mix. Samples were normalized to *Gapdh* expression. The sequences of the primers are listed in Supplemental Table 2.

RNA sequencing and data analysis

Total RNA from sorted ILC3s treated with IL-1β/IL-23 (10 ng/mL; Biolegend) in the presence or absence of SCF (100 ng/mL; Biolegend) for 6 hours at 37°C was extracted by using Direct-zol RNA Miniprep (Zymo Research), and used for the preparation of the sequencing library by TruSeq Stranded mRNA Library Prep Kit (Illumina) following the manufacturer's recommendations. Briefly, mRNA was purified from total RNA by oligo(dT)-coupled magnetic beads and fragmented into small pieces. The first-strand cDNA was synthesized using reverse transcriptase and random primers. After the generation of double-strand cDNA and adenylation on 3' ends of

DNA fragments, the adaptors were ligated. The products were enriched with PCR and purified with AMPure XP system (Beckman Coulter). The libraries were qualified by Qsep400 System (Bioptic Inc.) and Qubit 2.0 Fluorometer (Thermo Scientific) and then sequenced on Illumina NovaSeq 6000 system in Genomics, Taiwan. Raw reads were processed using fastp (v0.20.0) to perform read QC and adaptor trimming. Filtered reads were then aligned to the mouse reference genome (GRCm38) using HISAT2 (v2.1.0). Following this alignment, the software FeatureCounts (v2.0.1) in the Subread package was applied for gene expression quantification. Genes with <10 read counts in all samples were excluded. Normalization and differential gene expression analysis were performed by using the R package DESeq2, version 1.42.1 (3). The functional enrichment analysis of Gene Ontology (GO) terms and Kyoto Encyclopedia of Genes and Genomes (KEGG) pathways was performed using clusterProfiler, version 4.10.1 (4). Normalized counts generated by DESeq2 were used to perform gene set enrichment analysis (GSEA) by using GSEA software (v4.3.3, https://www.gsea-msigdb.org/gsea/).

For asthma microarray datasets, transcriptomic data for blood samples (5) and sputum samples (6) in the Unbiased Biomarkers for the Prediction of Respiratory Disease Outcomes (U-BIOPRED) Project were available from the NCBI GEO database (accession: GSE69683 and GSE76262).

For single cell RNAseq data analysis, KIT expression of human lung T cells and innate lymphoid cells were obtained from part of the Human Cell Atlas (7) and downloaded by using CELLxGENE data portal (https://cellxgene.cziscience.com/collections/62ef75e4-cbea-454e-a0ce-998ec40223d3). KITLG (SCF) and COL1A2 expression of human asthmatic lung cell atlas was obtained from part of the Human Cell Atlas (8) and downloaded from https://asthma.cellgeni.sanger.ac.uk. The dataset of SCF and Col1a2 expression in mouse lung

cells (9) were downloaded from https://tabula-muris.ds.czbiohub.org/ and analyzed in R (v4.2.1) using the Seurat package (v4.3.0).

BMMC culture and injection

Bone marrow-derived mast cells (BMMCs) were obtained as previously described (10). Briefly, bone marrow cells were isolated from the femurs and tibias of C57BL/6 mice and cultured in RPMI 1640 supplemented with 10% FBS, 50 μM 2-mercaptoethanol, IL-3, and SCF (all at 10 ng/mL) for at least five weeks. After five weeks of culture, BMMC purity was assessed by flow cytometry and the purity was ≥ 90% (Supplemental Figure 1D). For adoptive transfer of BMMCs, 10^7 BMMCs were intravenously injected into *Kit*$^{W\text{-}sh}$ mice on day 0, followed by three consecutive days (days 1–3) of intranasal IL-1β/IL-23 administration. Mice were sacrificed on day 4 for further analysis.

Supplemental References

1. Chang YJ, et al. Innate lymphoid cells mediate influenza-induced airway hyper-reactivity independently of adaptive immunity. *Nat Immunol.* 2011;12(7):631-8.
2. Fonseca W, et al. Group 2 innate lymphoid cells (ILC2) are regulated by stem cell factor during chronic asthmatic disease. *Mucosal Immunol.* 2019;12(2):445-56.
3. Love MI, et al. Moderated estimation of fold change and dispersion for RNA-seq data with DESeq2. *Genome Biol.* 2014;15(12):550.
4. Yu G, et al. clusterProfiler: an R package for comparing biological themes among gene clusters. *OMICS.* 2012;16(5):284-7.
5. Bigler J, et al. A Severe Asthma Disease Signature from Gene Expression Profiling of Peripheral Blood from U-BIOPRED Cohorts. *American Journal of Respiratory and Critical Care Medicine.* 2016;195(10).
6. Kuo CS, et al. T-helper cell type 2 (Th2) and non-Th2 molecular phenotypes of asthma using sputum transcriptomics in U-BIOPRED. *Eur Respir J.* 2017;49(2).
7. Dominguez Conde C, et al. Cross-tissue immune cell analysis reveals tissue-specific features in humans. *Science.* 2022;376(6594):eabl5197.
8. Vieira Braga FA, et al. A cellular census of human lungs identifies novel cell states in health and in asthma. *Nat Med.* 2019;25(7):1153-63.
9. Tabula Muris C, et al. Single-cell transcriptomics of 20 mouse organs creates a Tabula Muris. *Nature.* 2018;562(7727):367-72.
10. Qu J, et al. Oxidized CaMKII promotes asthma through the activation of mast cells. *JCI Insight.* 2017;2(1):e90139.
